# From wastewater to resistance: characterization of multidrug-resistant bacteria and assessment of natural antimicrobial compounds

**DOI:** 10.3389/fmicb.2025.1612534

**Published:** 2025-07-10

**Authors:** Mingyue Li, Angela Zhan, Tahira Tasneem Rahman, Tao Jiang, Liyuan Hou

**Affiliations:** ^1^Utah Water Research Laboratory, Logan, UT, United States; ^2^Department of Civil and Environmental Engineering, Utah State University, Logan, UT, United States; ^3^School of Resources and Environmental Engineering, Shandong University of Technology, Zibo, China; ^4^Logan High School, Logan, UT, United States; ^5^Department of Environmental and Sustainable Engineering, University at Albany, State University of New York, Albany, NY, United States

**Keywords:** natural compounds, curcumin, emodin, antibiotic-resistant bacteria, wastewater treatment plant, whole-genome sequencing

## Abstract

The development and spread of antibiotic resistance in wastewater pose significant threats to both the environment and public health. Bacteria harboring multiple antibiotic resistance genes (ARGs), including those associated with horizontal gene transfer (HGT), can serve as persistent reservoirs and vectors for antimicrobial resistance in natural ecosystems. In this study, nine antibiotic-resistant bacterial strains (U1–U9) were isolated from a wastewater treatment plant (WWTP) effluent. The isolates were identified using 16S rRNA gene sequencing and whole-genome sequencing (WGS), and their antibiotic susceptibility profiles were evaluated. All isolates exhibited resistance to multiple antibiotics, and WGS revealed that U1, U2, U4, and U7 harbored diverse ARGs, including *β*-lactamase genes, efflux pumps, and resistance determinants for sulfonamides, tetracyclines, and, quinolones, confirming the presence of multidrug-resistant bacteria in WWTP effluent. Phylogenetic analysis classified them into *Microbacterium* spp. (Actinobacteria), *Chryseobacterium* spp. (Bacteroidetes), *Lactococcus lactis* spp. (Firmicutes), and *Psychrobacter* spp. (Proteobacteria). To explore mitigation strategies, eleven natural compounds were screened for their effects on cell growth, biofilm formation, and motility in selected multi-drug-resistant bacteria. Among the tested compounds, curcumin and emodin showed the most consistent inhibitory activity, particularly against *Microbacterium* spp. strains U1 and U2, and *Lactococcus lactis* sp. U4. In contrast, *Chryseobacterium* sp. U7, a Gram-negative strain, exhibited strong resistance to all tested natural compounds, highlighting the challenge of controlling Gram-negative ARBs in wastewater settings. These findings underscore the environmental risks posed by multidrug-resistant and HGT-associated ARG-harboring bacteria in WWTP effluent. They also demonstrate the potential of natural products, such as curcumin and emodin, as alternative or complementary agents for mitigating antibiotic resistance in water systems.

## 1 Introduction

Antibiotics have made remarkable strides in treating infections caused by pathogenic bacteria, playing a crucial role in improving human, animal, and plant health. However, the widespread misuse and overuse of antibiotics have led to the emergence of antibiotic resistance (AR), which has been recognized as a major global health threat by the World Health Organization (WHO). AR is a natural evolutionary process that occurs when antibiotic-resistant bacteria (ARB) harboring antibiotic resistance genes (ARGs) are exposed to antibiotics. Under selective pressure, susceptible bacteria are inhibited or killed, while those with intrinsic resistance (through naturally occurring ARGs) or those acquiring resistance via horizontal gene transfer gain a survival advantage (Prestinaci et al., 2015). Prolonged and excessive antibiotic use has significantly contributed to the emergence, persistence, and rapid dissemination of ARB and ARGs, intensifying the spread of AR (McEwen and Collignon, 2018; Singh et al., 2024). The phenomenon is further complicated by the horizontal transfer of resistance genes among bacterial communities (McEwen and Collignon, 2018). The antibiotic resistance maps illustrate the global distribution and prevalence of AR (OneHealthTrust, 2023). Today, AR is not only one of the most critical global health crises but also poses significant economic, social, and labor-related challenges (Soni et al., 2022). According to the WHO, antibiotic resistance is accelerating, and effective treatment options are rapidly diminishing (Lawe-Davies and Bennett, 2017).

A significant proportion of administered antibiotics is excreted unmetabolized in urine and feces and discharged into drainage systems (Uyaguari-Díaz et al., 2018). As a result, substantial amounts of antibiotics persist in anthropogenic environments such as wastewater treatment plants (WWTPs) and sewage systems. These antibiotics exert selective pressure on various bacteria and genes, including ARB and ARGs, thereby enhancing AR in WWTPs. WWTPs serve as hotspots for AR proliferation, providing ideal conditions, such as high microbial load, abundant organic compounds, nutrients, biocides, and antibiotics, for the survival and spread of ARB and ARGs (Uluseker et al., 2021). Numerous studies have demonstrated that sewage of the WWTPs is the foremost anthropogenic pool of AR, supporting the persistence and propagation of ARB and ARGs (WHO, 2017). Notably, the concentration of ARGs in WWTP effluent is often higher than in natural rivers. Consequently, the discharge of WWTP effluent into rivers facilitates the environmental dissemination of ARGs. Many rivers with elevated levels of antibiotics, ARB, and ARGs are directly impacted by urban wastewater inputs (Wu et al., 2023). Therefore, WWTPs serve as critical junctions linking human activities and the environment, facilitating the horizontal transfer of ARGs between environmental microorganisms and clinically relevant pathogens. Additionally, an increasing number of bacterial strains are exhibiting resistance to a broader range of antibiotics. Some of these are multi-resistant bacteria that carry multiple resistance genes, rendering them resistant to all, or nearly all, approved antimicrobial agents available for treating their infections (Prestinaci et al., 2015). Given this role, it is essential to investigate ARB in WWTP effluents to identify multidrug-resistant bacteria, explore approaches to mitigate their spread, and elucidate their potential to facilitate horizontal gene transfer of antibiotic resistance.

The mechanisms and dissemination pathways of AR are highly complex, involving diverse microbial hosts, resistance genes, and mobile genetic elements. Addressing this complexity requires the integration of advanced molecular tools, among which microbial whole-genome sequencing (WGS) has emerged as a transformative approach. Compared to traditional typing methods, WGS offers significantly higher resolution, enabling detailed characterization of bacterial strains, including their phylogenetic relationships, resistance gene profiles, and mobile genetic elements such as plasmids, integrons, and transposons (Brlek et al., 2024). This level of detail facilitates a better understanding of the origin, evolution, and transmission dynamics of multidrug-resistant bacteria. Previous studies have applied whole-genome sequencing to analyze multi-drug-resistant strains isolated from WWTPs, suggesting that it is an essential tool for providing rapid and comprehensive data on resistance genes, disclosing potential molecular mechanisms of antibiotic resistance, and revealing not only the presence of clinically relevant resistance genes (e.g., *bla*, *mcr*, *tet*, *van*) but also the potential for horizontal gene transfer among environmental and pathogenic microbes (Surleac et al., 2020; Al-Mustapha et al., 2025). WGS data can also be integrated with metagenomic analyses and bioinformatics pipelines to monitor resistance trends over time and across treatment stages (Liang et al., 2023; Rout et al., 2024). This makes WGS a critical tool for both surveillance and the development of targeted mitigation strategies. As antibiotic resistance continues to pose a global health threat, the integrating WGS data with metagenomic and bioinformatics tools is effective for tracking and understanding resistance trends in WWTPs.

While tools such as whole-genome sequencing are critical for monitoring resistance and informing mitigation strategies, technological solutions alone may not be sufficient to curb the rise of antimicrobial resistance. This highlights the urgent need to explore alternative approaches to antibiotic therapy. Consequently, some common infections have become increasingly difficult or even impossible to treat, leading to prolonged illness, higher mortality rates, and rising healthcare costs (Wozniak et al., 2019; AlSheikh et al., 2020). In addition, the discovery and development of new antibiotics has declined over the last decades, resulting in an “antibiotics crisis,” largely because the rapid emergence of resistance often shortens the effective lifespan of new antibiotics, discouraging pharmaceutical investment in this field (Cook and Wright, 2022). More importantly, the widespread use of high doses of antibiotics not only promotes AR development but also poses serious environmental risks. Therefore, the research and development of antimicrobial strategies have become urgent. One promising approach involves the exploration and application of natural compounds, which are considered safer and more sustainable than conventional synthetic antibiotics (Naiel et al., 2023; Arrigoni et al., 2024). These natural antimicrobials, such as polyphenols, alkaloids, and lanthipeptides, are often derived from sources like plants and marine organisms. Despite their potential, the application of these natural compounds faces several challenges, including low concentrations of active constituents in natural extracts, unstable chemical structures, and limited efficacy and shelf life. Among the most pressing challenges is the need to evaluate the effectiveness of natural compounds on multi-drug-resistant bacteria from different sources or environments. For instance, the effects of natural compounds on antibiotic-resistant bacteria from urban wastewater effluent remain largely unexplored. In addtion, the occurrence of compounds like curcumin, quercetin, and emodin in wastewater and surface waters is poorly documented.

While previous studies have examined antibiotic resistance in clinical pathogens or used metagenomic approaches to survey resistance genes in wastewater, few have investigated antibiotic-resistant bacteria from wastewater effluent at the strain level using whole-genome sequencing. Even fewer have evaluated the antimicrobial effects of natural compounds on these environmentally derived multidrug-resistant bacteria. Thus, the aim of the present study was to (i) isolate antibiotic resistance bacteria from the effluent of an urban wastewater plant and test their multidrug resistance; (ii) identify and characterize the isolated bacteria using 16S rRNA gene sequencing and whole-genome sequencing, with a focus on uncovering the underlying genetic mechanisms responsible for their multidrug resistance; and (iii) screen natural compounds as alternatives to antibiotics for controlling multidrug-resistant bacteria. These objectives provide critical insights into the prevalence and characteristics of multidrug-resistant bacteria in treated wastewater, highlighting the potential risks associated with environmental dissemination of antibiotic resistance. By identifying effective natural compounds, this research also contributes to the development of sustainable and environmentally friendly alternatives to synthetic antibiotics. The findings can inform future mitigation strategies and public health policies aimed at curbing the spread of antibiotic resistance from anthropogenic sources to the broader environment.

## 2 Methods and materials

### 2.1 Isolation of antibiotic-resistant bacteria from the WWPT effluent

Effluent samples were collected from a local WWTP located in Logan, Utah, USA. Luria–Bertani (LB) agar (Fisher Scientific, PA, USA) plates were prepared with or without antibiotics, including sulfamethoxazole, carbenicillin, erythromycin, kanamycin, nalidixic acid, and tetracycline (each at 50 μg/mL); chloramphenicol (25 μg/mL); and colistin (1 and 4 μg/mL). The initial selection using sulfamethoxazole served as a representative screening step for sulfonamide resistance. A 150 μL aliquot of the effluent sample was inoculated onto the LB agar with sulfamethoxazole and spread evenly on the surface with a sterile cotton swab. Plates were incubated at 28°C for 2–3 days. Colonies exhibiting distinct morphologies, sizes, or pigmentation were carefully selected and re-streaked onto fresh LB plates containing sulfamethoxazole for purification. After a second incubation period of 2–3 days at 28°C, nine sulfamethoxazole-resistant colonies (designated U1-U9) were obtained. To further assess multidrug resistance, these isolates were tested for growth on LB agar plates supplemented with additional antibiotics as listed above, using the same plating and incubation procedures. Growth was scored qualitatively based on colony formation. This approach provides a high-throughput and effective means to identify environmental bacteria with high-level resistance and has been widely adopted in environmental screening studies (Billet et al., 2021; Ozaktas et al., 2012). Although this method does not follow standardized interpretive protocols such as CLSI or EUCAST, it enables detection of isolates with robust antibiotic tolerance. In this study, MDR was classified according to the definitions proposed by Magiorakos et al. (2012), where MDR is defined as resistance to at least one agent in three or more antimicrobial classes.

### 2.2 Identification of the antibiotic-resistant bacteria through 16S rRNA gene sequencing

Distinct bacterial colonies of U1-U9 were picked and cultured in LB broth for 2–3 days at 28°C to obtain sufficient biomass for DNA extraction. Microbial DNA was extracted using a *Quick*-DNA Fungal/Bacterial Miniprep Kit (Zymo Research, USA) according to the manufacturer’s instructions. The extracted and purified DNA was stored at −20°C before use. The 16S rRNA gene was amplified with primers 27F (AGAGTTTGATCCTGGCTCAG) and 1492R (GGCTACCTTGTTACGACTTC) (Oldham and Duncan, 2012) in 20 μL PCR reaction volume. Each 20-μL mixture contained 0.2 μL of Phusion® High-Fidelity DNA Polymerase (Thermo Fisher Scientific, USA), 4 μL of 5 × buffer (Thermo Fisher Scientific, USA), 0.6 μL of dimethyl sulfoxide (DMSO) (Thermo Fisher Scientific, USA), 0.4 μL of dNTPs (10 mM each, Thermo Fisher Scientific, USA), 1 μL of each primer (10 μM), 0.2 μL of DNA template and 12.6 μL of deionized water. For the negative control, deionized water was added in an equivalent amount instead of the DNA template. PCR was performed in a T100™ Thermal Cycler (Bio-Rad, USA). The conditions were: initial denaturation at 98°C for 30 s; followed by 30 cycles of denaturation at 98°C for 10 s, annealing at 57°C for 30 s, and extension at 72°C for 30 s; with a final extension at 72°C for 5 min. The final PCR product was viewed in agarose gel electrophoresis and visualized and photographed by a UV transilluminator. The objective gel was cut and extracted for 16S rRNA gene fragments using a GeneJET Gel Extraction Kit (Thermo Fisher Scientific, USA) according to the manufacturer’s instructions. Then, the 16S rRNA gene fragments were sequenced at Eton Bioscience, Inc. via Sanger Sequencing. The obtained sequences were analyzed using BLAST to find similarities with the known strains. The phylogenetic tree with neighbor-joining analysis was reconstructed using the MEGA 11 program (Kumar et al., 2018). The obtained 16S rRNA sequences were deposited in the GenBank with accession numbers SUB15239090 (PV459613-PV459620).

### 2.3 Whole genome sequencing of multiple-antibiotic-resistant bacteria

Genomic DNA from four selected multiple-antibiotic-resistant bacteria (U1, U2, U4, and U7) was extracted and submitted to Molecular Research LP (MR DNA, www.mrdnalab.com, TX, USA). A total of 50 ng of high-quality DNA from each isolate was used for library preparation using the SMRTbell® libraries according to the manufacturer’s protocol (Pacific Biosciences, CA, USA), including DNA shearing, end-repair, adapter ligation, and size selection. Sequencing was performed on the PacBio RS II platform using single-molecule real-time (SMRT) sequencing technology. High-fidelity (HiFi) long-read sequences were generated, producing genome assemblies approximately 4–5 Mb in length with greater than 75 × coverage per isolate. Genome assembly was carried out using NGEN (DNASTAR, USA), and annotation was performed using the Bactopia pipeline. Each genome output included standard file formats such as assembled contigs (.fasta), annotated genome files (.gbff, .embl, .gff3), protein and nucleotide sequences (.faa, .fna), gene tables (.tsv), metadata (.json), and visualizations (.png, .svg). The annotated genomes were further analyzed to identify antibiotic resistance genes, virulence-associated factors, and to construct phylogenetic relationships. All sequences were deposited in the GenBank under BioProject accession PRJNA1246262.

ARGs were identified using the Comprehensive Antibiotic Resistance Database (CARD), a curated and regularly updated resource that integrates sequence data, resistance mechanisms, and antibiotic classes via its Antibiotic Resistance Ontology (ARO). Amino acid sequences from the isolates were uploaded to the CARD online BLAST tool (https://card.mcmaster.ca/analyze/blast) for annotation. The annotated gene hits were filtered, categorized by resistance type, and summarized by gene family. To identify virulence-associated genes, amino acid sequences of the assembled genomes were compared against the Virulence Factor Database (VFDB), which curated virulence factors from pathogenic bacteria, including *Chlamydiae* and *Rickettsiae*. As of November 2017, VFDB contained 17,896 virulence factors from 926 strains across 74 genera, encompassing 30,178 non-redundant VF-related genes. BLASTP was used to align amino acid sequences to the VFDB database, with an e-value threshold set to 1e^−5^ to ensure stringency.

### 2.4 Effect of natural compounds on cell growth of multiple-antibiotic-resistant bacteria

A single bacterial colony from U1, U2, U4, and U7 was inoculated into 4 mL of LB broth supplemented with 2% glucose and incubated at 28°C with continuous shaking at 250 rpm overnight to prepare an active bacterial suspension. Eleven natural compounds were tested for their antimicrobial activity. These included berberine, chlorflavonin, chrysin, curcumin, emodin, hesperidin, naringin, quercetin, resveratrol, rutin, and 2′-hydroxyflavone (Supplementary Figure S1). These compounds, originally derived from plants or microbes, are primarily members of the polyphenols family, characterized by aromatic rings and phenolic hydroxyl groups, with the exception of berberine (an alkaloid) and emodin (an anthraquinone). All compounds were dissolved in DMSO before use. Antimicrobial assays were performed in sterile 96-well flat-bottomed microtiter plates. Each well was filled with 150 μL of bacterial suspension (adjusted to ~1 × 10^6^ CFU/mL after the overnight culture) and 4 μL of compound solution. For each natural compound, two different concentrations, 13.33 and 26.67 μg/mL, were tested. While their antimicrobial activity has been demonstrated *in vitro*, background concentrations of these compounds in environmental samples are rarely monitored. Thus, the concentrations used in this study (13.33 and 26.67 μg/mL) were chosen to assess biological efficacy rather than reflect environmental exposure levels. Wells containing 4 μL of DMSO without any compound served as the negative control. All treatments were conducted in triplicate. Plates were incubated at 28°C with shaking at 250 rpm. Bacterial growth was monitored by measuring the optical density at 600 nm (OD_600_) using a SpectraMax iD3 Plate Reader (Molecular Devices, CA, USA) at 0, 12, 24, and 48 h. Changes in OD_600_ values were used to assess the inhibitory effects of the natural compounds over time.

### 2.5 Effect of natural compounds on biofilm formation of multiple-antibiotic-resistant bacteria

Biofilm inhibition assay was performed as described previously (Alam et al., 2020). As in Section 2.4, single colonies of U1, U2, U4, and U7 were inoculated into LB broth with 2% glucose and incubated at 28°C with shaking at 250 rpm overnight. The resulting bacterial suspensions were then used for biofilm inhibition assays. The same eleven natural compounds described in Section 2.4 were used at two concentrations (13.33 and 26.67 μg/mL) to evaluate their effects on biofilm formation. All compounds were dissolved in DMSO, and wells containing 4 μL of DMSO without any compound served as negative controls. Subsequently, 96-well plates were incubated at 28°C without shaking for 20 h. After incubation, the content of each well was discarded, and the wells were washed three times with sterile double distilled water. Plates were blotted dry with paper towels and placed in a 60°C oven for 10 min to fix the biofilms. Each well was stained with 50 μL of 0.4% crystal violet solution for 15 min at room temperature. Excess stain was removed by rinsing the wells four times with water. After drying again at 60°C for 10 min, 100 μL of 30% acetic acid was added to each well and incubated for 10 min at room temperature to solubilize the dye. Biofilm biomass was quantified by measuring the optical density at 550 nm (OD_550_) using the microplate reader.

### 2.6 Effect of selected natural compounds on motility of multiple-antibiotic-resistant bacteria

A single colony of strains U1, U2, and U4 was inoculated into 3 mL of LB broth supplemented with 2% glucose and incubated at 28°C with continuous shaking at 250 rpm overnight. U7 was not included in the motility assay, as it exhibited minimal sensitivity to the selected natural compounds in both cell growth and biofilm formation assays. To ensure the cells were in an active growth phase suitable for motility assessment, 20 μL of the overnight culture was transferred into a fresh tube containing 3 mL of LB broth with 2% glucose and incubated under the same conditions for an additional 16 h. The OD₆₀₀ was measured, and cultures were adjusted to approximately 10^6^ CFU/mL using LB broth containing 2% glucose. Swimming motility was assessed on LB agar plates containing 0.3% agar (Ha et al., 2014) while swarming motility was tested on LB agar plates supplemented with 0.5% glucose and 0.5% agar to support coordinated surface movement (Kearns, 2010). For both assays, 5 μL of the diluted bacterial suspension containing a selected natural compound was inoculated at the center of each plate. Based on the results from the cell growth and biofilm inhibition assays, curcumin and emodin were selected for U1; curcumin, emodin, and chlorflavonin were tested with U2; and emodin was used for U4. Colony diameters were recorded at various time points: 0, 24, 28, 36, 48, 60, and 72 h using ImageJ software (NIH, MD, USA).

### 2.7 Statistical analysis and data visualization

Different statistical and computational methods were applied based on the nature of the experimental data. For quantitative assays such as cell growth, swimming motility, and swarming motility, paired-samples t-tests were used to assess statistical significance between two matched treatment groups. For biofilm formation, paired comparison plots were employed to visualize and compare biofilm biomass under different treatment conditions. Metagenomic and functional annotation data were processed and visualized using the ggplot2 package in R. Phylogenetic analysis was performed using OrthoFinder, which constructs genome-scale phylogenies based on protein sequence similarity across isolates. DIAMOND (v2.0.7) was used as the alignment engine within OrthoFinder. The resulting tree files were visualized and annotated using iTOL (Interactive Tree Of Life; https://itol.embl.de/), where custom metadata, labels, and color schemes were applied to enhance biological interpretability.

## 3 Results

### 3.1 Isolation and characterization of nine antibiotic-resistant bacteria from WWTP effluent

Nine bacterial strains (U1-U9) were isolated from the final effluent of a WWTP, all exhibiting resistance to two or multiple antibiotics (Table 1). Strain U2 showed the broadest resistance profile, demonstrating tolerance to all eight tested antibiotics. However, its growth was notably reduced in the presence of chloramphenicol and tetracycline, suggesting a weaker resistance to these compounds. Strains U1 and U7 exhibited resistance to six of the eight antibiotics tested, indicating a high level of multidrug resistance. Interestingly, all strains except U9 showed robust growth on LB agar plates supplemented with colistin at both 1 and 4 mg/L. Strain U9 exhibited very limited survival, with only one or two colonies observed. Colistin is often regarded as a “last resort” antibiotic for treating infections caused by multidrug-resistant Gram-negative bacteria (Mondal et al., 2024). The widespread resistance observed suggests that such resistance may already be emerging and spreading in wastewater microbial communities. To assess the reproducibility of this observation, a new set of influence and effluent samples were collected and plated on LB agar plate with 1 μg/mL colistin. The influent sample produced numerous colistin-resistant colonies, while only two colonies were detected in the effluent (Supplementary Figure S2). These results indicate that the selected WWTP process effectively removes most colistin-resistant bacteria; however, some persist in the effluent. Additional treatment steps may be necessary to ensure the water is safe for environmental discharge.

**Table 1:** Summary of the antibiotic resistance of the nine isolated strains.

Strain		Antibiotic resistance							
		Sulfametho-xazole	Carbenicillin	Chloramphenicol	Erythromycin	Kanamycin	Nalidixic acid	Tetracycline	Colistin
U1	*Microbacterium* sp.	√	√	-	√	√	√	-	√
U2	*Microbacterium* sp.	√	√	√^−^	√	√	√	√^−^	√
U3	*Chryseobacterium* sp.	√^−^	√	-	-	√	-	-	√
U4	*Lactococcus lactis* sp.	√	-	-	-	-	√	-	√
U5	*Chryseobacterium* sp.	√^−^	√	√^−^	-	√	-	-	√
U6	*Lactococcus lactis* sp.	√	-	-	-	-	√	-	√
U7	*Chryseobacterium* sp.	√	√	√	√	√	-	-	√
U8	*Lactococcus lactis* sp.	√	-	-	-	-	√	-	√
U9	*Psychrobacter* sp.	√	-	-	-	-	-	-	√^−^

√, well growth; √^−^, weak growth; −, no growth.

### 3.2 Identification of antibiotic-resistant bacteria through 16S rRNA gene sequencing

**Figure 1:**
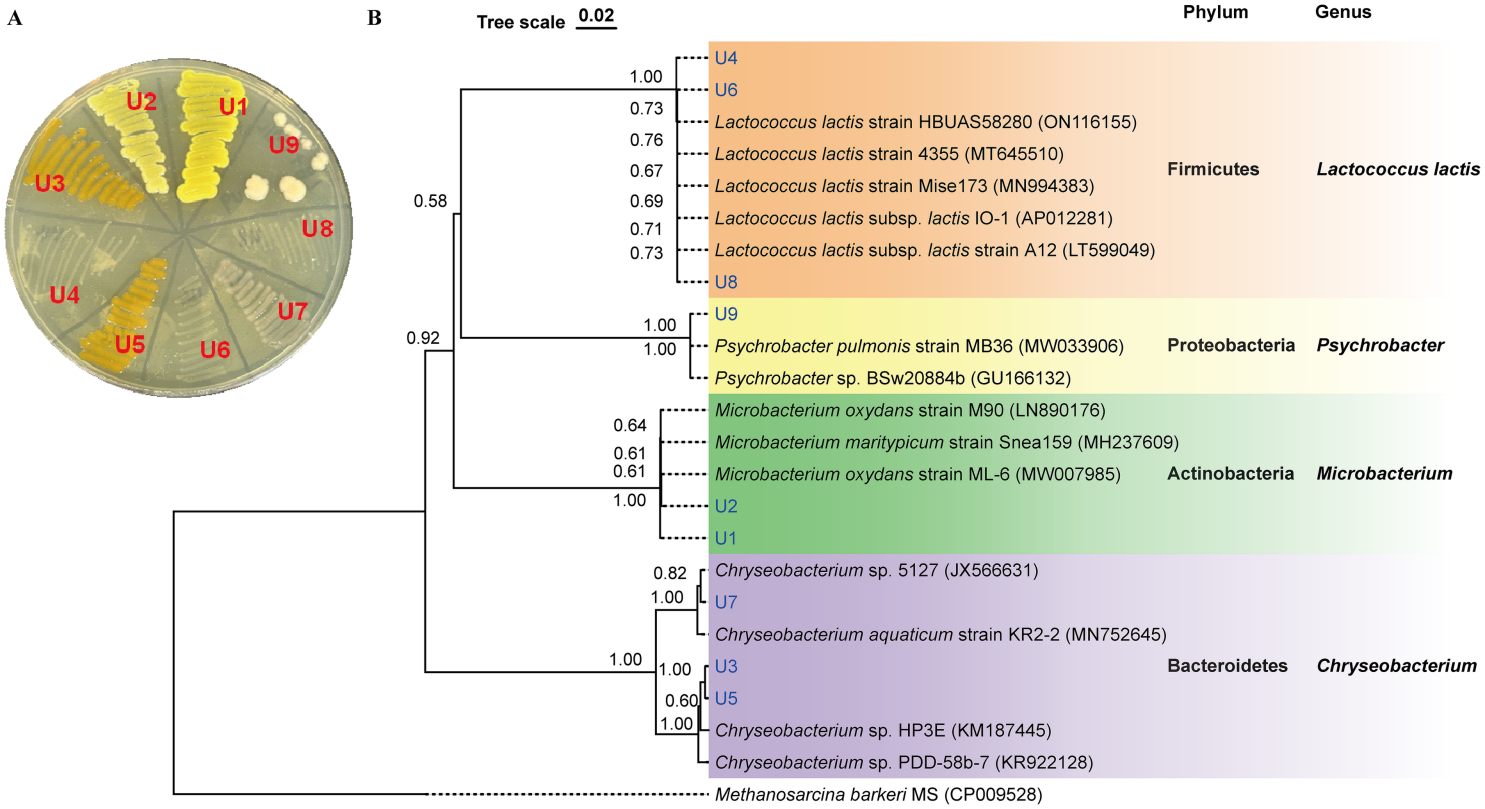
Identification of isolated antibiotic-resistant bacteria from WWTP effluent. **(A)** Colony morphology of isolates U1-U9 on LB agar. **(B)** Phylogenetic tree based on 16S rRNA gene sequences of U1-U9. The tree was constructed using neighbor-joining with *p*-distance model. Bootstrap values (1,000 replicates) are shown at the nodes. The scale bar corresponds to 0.02 substitutions per nucleotide position.

The colony morphology of strains U1-U9 is shown in Figure 1A. Among them, four isolates (U1, U2, U3, and U5) exhibited noticeable pigmentation. To identify the bacterial isolates and determine their phylogenetic relationships, 16S rRNA gene sequencing was performed. BLAST analysis revealed that the sequences shared high similarity (99.02 to 100%) with known bacterial strains (Supplementary Table S1). The antibiotic-resistant isolates belonged to four major bacterial phyla: Firmicutes, Bacteroidetes, Actinobacteria, and Proteobacteria. Specifically, U1 and U2 were phylogenetically clustered as *Microbacterium* spp. (Actinobacteria); U3, U5 and U7 were classified as *Chryseobacterium* spp. (Bacteroidetes); U4, U6 and U8 were closely related to *Lactococcus lactis* spp. (Firmicutes); and U9 was identified as *Psychrobacter* sp. (Proteobacteria) (Figure 1B). The predominance of *Chryseobacterium* spp. and *Lactococcus lactis* spp. suggests that these taxa may play key roles in the persistence or spread of resistance within wastewater systems.

### 3.3 Whole genome sequencing and analysis of four multiple-antibiotic-resistant bacteria

**Figure 2:**
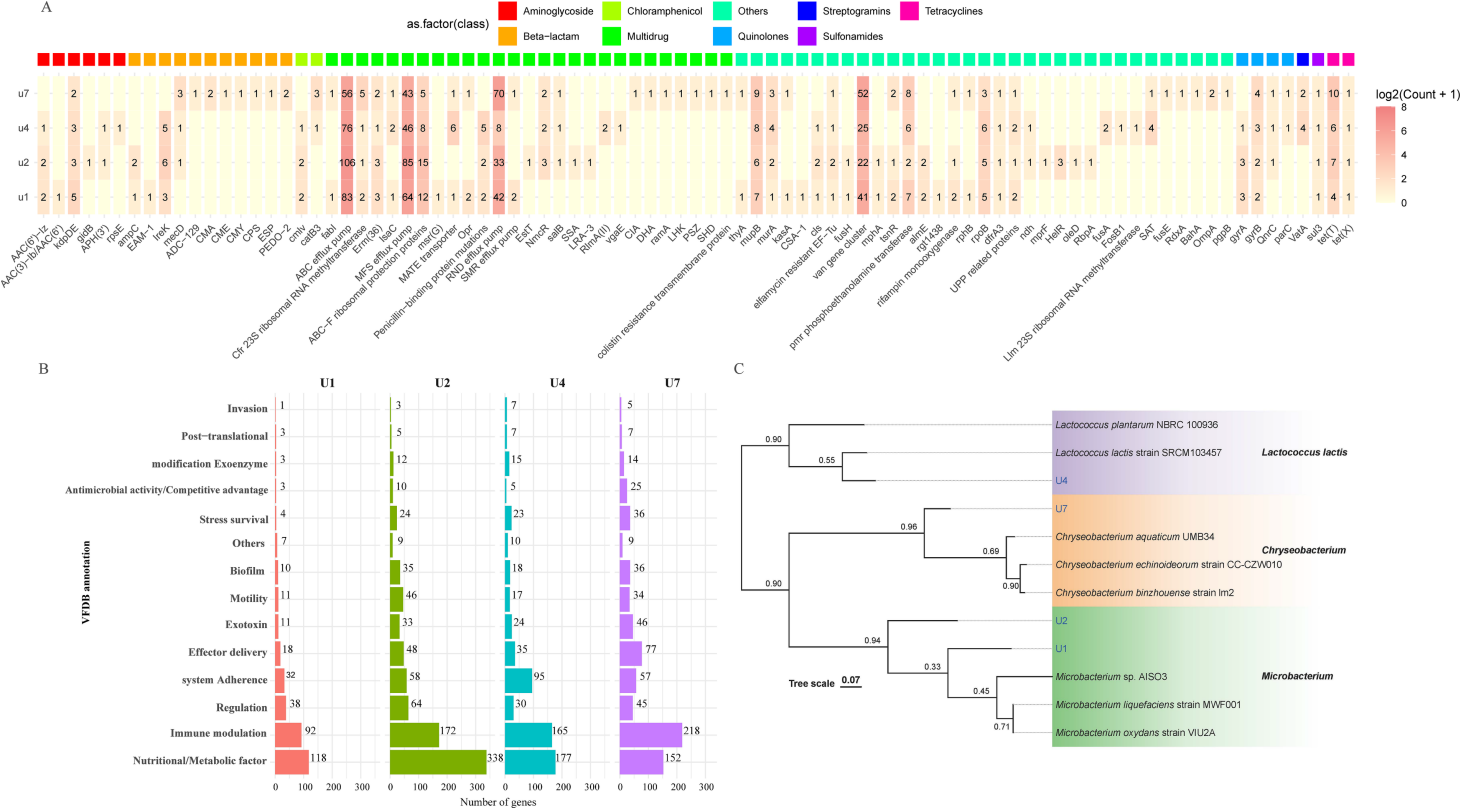
Whole-genome-based analysis of four multiple-antibiotic-resistant bacterial strains. **(A)** Distribution and classification of ARGs identified in U1, U2, U4, and U7. **(B)** Functional classification of predicted virulence factors based on VFDB annotations for U1, U2, U4, and U7. **(C)** Phylogenetic tree constructed from whole-genome sequences of U1, U2, U4, and U7 using genome-scale comparisons. Bootstrap values are indicated at branch nodes.

To better understand the genomic features and resistance mechanisms of the antibiotic-resistant bacteria, four representative strains were chosen for whole genome sequencing: *Microbacterium* sp. U1, *Microbacterium* sp. U2, *Lactococcus lactis* sp. U4, and *Chryseobacterium* sp. U7. The genome maps are shown in Supplementary Figure S3. The genome size of U1 was 3,552,263 base pairs (bp), with a GC content of 68.2%, while U2 has a genome size of 4,137,647 bp and a GC content of 68.5%. These results align with the reported average genome size and GC content of *Microbacterium* species, which typically have genome size above 3.5 Mb, and GC content around 70% (Corretto et al., 2020). For U4 and U7, the genome sizes were 2,485,453 bp and 4,464,581 bp, respectively, with GC contents of 35.2 and 33.8%. These measurements are consistent with reported genomes of *Lactococcus lactis* and *Chryseobacterium* species (Matu et al., 2019; Wan et al., 2021), respectively. Further analysis of the annotated genes indicated that these antibiotic-resistant bacteria contain a variety of genes contributing to the resistance against various antibiotics, including aminoglycoside, *β*-lactam, chloramphenicol, quinolones, streptogramins, sulfonamides, and tetracyclines (Figure 2A). U2 exhibited the highest diversity and abundance of ARGs across nearly all classes, consistent with its phenotype resistance to all eight antibiotics tested. U2 also showed the highest number of virulence genes across categories (Figure 2B), particularly in adherence, motility, and immune modulation, which may contribute to its fitness and persistence in the WWTP environment. U4 and U7 also displayed notable virulence-related functions, especially in biofilm formation, nutrient acquisition, and stress survival. U1, while genetically simpler, still carried genes related to adherence, immune evasion, and biofilm formation, suggesting potential pathogenic traits. Phylogenetic analysis of the four strains based on whole genome sequencing was in accordance with the 16S rRNA gene sequencing results (Figure 2C). *Microbacterium* sp. U2 is the most genetically equipped strain, harboring the highest number of ARGs and virulence genes, making it a potential environmental reservoir for antimicrobial resistance.

### 3.4 Effects of natural compounds on cell growth and biofilm formation of multiple-antibiotic-resistant bacteria

**Figure 3:**
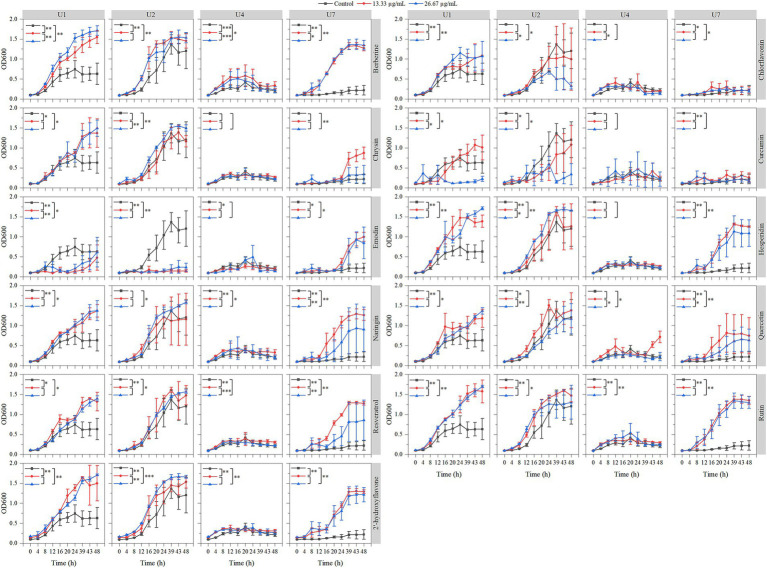
Effects of 11 natural compounds on cell growth of multiple-antibiotic-resistant bacteria (i.e., U1, U2, U4, and U7). The variation between different treatments was calculated by a paired- samples *t*-test; *, *p* < 0.05; **, *p* < 0.01; ***, *p* < 0.001.

The effects of 11 natural compounds on cell growth were evaluated by measuring OD_600_ for four multiple-antibiotic-resistant bacteria: *Microbacterium* sp. U1, *Microbacterium* sp. U2, *Lactococcus lactis* sp. U4, and *Chryseobacterium* sp. U7. For *Microbacterium* sp. U1 (Figure 3; Table 2), curcumin significantly inhibited bacterial growth at 26.67 μg/mL (*p* < 0.05) but not at 13.33 μg/mL. Emodin significantly reduced growth at both concentrations (*p* < 0.05). In contrast, the remaining nine compounds significantly promoted the cell growth at both concentrations (*p* < 0.05). For *Microbacterium* sp. U2 (Figure 3; Table 2), curcumin and emodin significantly inhibited growth at both concentrations (*p* < 0.05), while chlorflavonin showed inhibitory activity only at 26.67 μg/mL (*p* < 0.05). Several compounds, including berberine, hesperidin, resveratrol, rutin, and 2′-hydroxyflavone, significantly promoted growth at both concentrations (*p* < 0.05). Chrysin and naringin enhanced growth only at 26.67 μg/mL, whereas quercetin exhibited a significant stimulatory effect at 13.33 μg/mL (*p* < 0.05). For *Lactococcus lactis* sp. U4, emodin and quercetin significantly inhibited growth at 13.33 μg/mL and 26.67 μg/mL, respectively (*p* < 0.05). No significant differences in growth were observed between control group and treatment groups for chlorflavonin, chrysin, curcumin, and hesperidin (*p* > 0.05). Conversely, berberine, naringin, rutin, and 2′-hydroxyflavone significantly promoted growth at both concentrations, while resveratrol had a significantly growth-promoting effect at 13.33 μg/mL (*p* < 0.05). For *Chryseobacterium* sp. U7, none of the tested natural compounds showed inhibitory effects on growth. All compounds, except 13.33 μg/mL chrysin and 26.67 μg/mL curcumin, significantly promoted growth (*p* < 0.05). Overall, emodin demonstrated the strongest inhibitory activity against cell growth across multiple strains, followed by curcumin. *Chryseobacterium* sp. U7 showed exceptional growth resilience, with all compounds either enhancing growth or having no significant inhibitory effect. This may reflect differences in cell wall structure and permeability, as U1, U2, and U4 are Gram-positive, whereas U7 is Gram-negative, suggesting that the tested natural compounds may be more effective against Gram-positive bacteria. In addition, future studies should explore a broader range of concentration gradients for each compound to better determine their dose-dependent effects.

**Table 2:** The effect of 11 natural compounds on cell growth and biofilm formation of multiple-antibiotic-resistant bacteria.

Natural compound	Tested concentration	Cell growth				Biofilm formation			
		U1	U2	U4	U7	U1	U2	U4	U7
Berberine	13.33 μg/mL	+**	+**	+***	+**	/	/	**↑**	/
	26.67 μg/mL	+**	+**	+*	+**	/	/	/	/
Chlorflavonin	13.33 μg/mL	+**	/	/	+*	**↑**	**↑**	**/**	**/**
	26.67 μg/mL	+**	**-** *	/	+*	**↑**	**↑**	**↑**	**↑**
Chrysin	13.33 μg/mL	+*	/	/	/	**/**	/	**↑**	**↑**
	26.67 μg/mL	+*	+**	/	+**	**↑**	**/**	**↑**	**/**
Curcumin	13.33 μg/mL	/	**-** *	/	+**	**/**	↓	**↑**	**/**
	26.67 μg/mL	**-** *	**-** *	/	/	↓	↓	↓	/
Emodin	13.33 μg/mL	**-** **	**-** **	**-** *	+*	**/**	↓	**/**	**/**
	26.67 μg/mL	**-** *	**-** **	/	+*	**/**	↓	**/**	**/**
Hesperidin	13.33 μg/mL	+**	+**	/	+**	**/**	**/**	**/**	**/**
	26.67 μg/mL	+**	+**	/	+**	**/**	**/**	**/**	**/**
Naringin	13.33 μg/mL	+**	/	+**	+**	**/**	**/**	**/**	**/**
	26.67 μg/mL	+*	+*	+*	+**	**/**	**/**	**/**	**/**
Quercetin	13.33 μg/mL	+**	+*	/	+**	**/**	↓	**/**	**/**
	26.67 μg/mL	+*	/	**-** *	+**	**↑**	↓	**↑**	**/**
Resveratrol	13.33 μg/mL	+*	+**	+**	+**	**/**	**/**	**/**	**/**
	26.67 μg/mL	+*	+*	/	+**	**/**	**/**	**/**	**/**
Rutin	13.33 μg/mL	+**	+**	+**	+**	**/**	**/**	**↑**	**/**
	26.67 μg/mL	+**	+*	+**	+**	**/**	**/**	**/**	**/**
2′-hydroxyflavone	13.33 μg/mL	+**	+**	+**	+**	**↑**	**/**	**↑**	**/**
	26.67 μg/mL	+**	+***	+**	+**	**↑**	**/**	**↑**	**/**

For cell growth: (−), inhibition; (+), promotion; (*), *P* < 0.05; (**), *P* < 0.01; (***), *P* < 0.001; (/), no effect (no significant difference compared with control group). For biofilm formation: (↓), significant inhibition (*P* < 0.05); (**↑**), significant promotion (*P* < 0.05); (/), no effect (no significant difference compared with control group).

**Figure 4:**
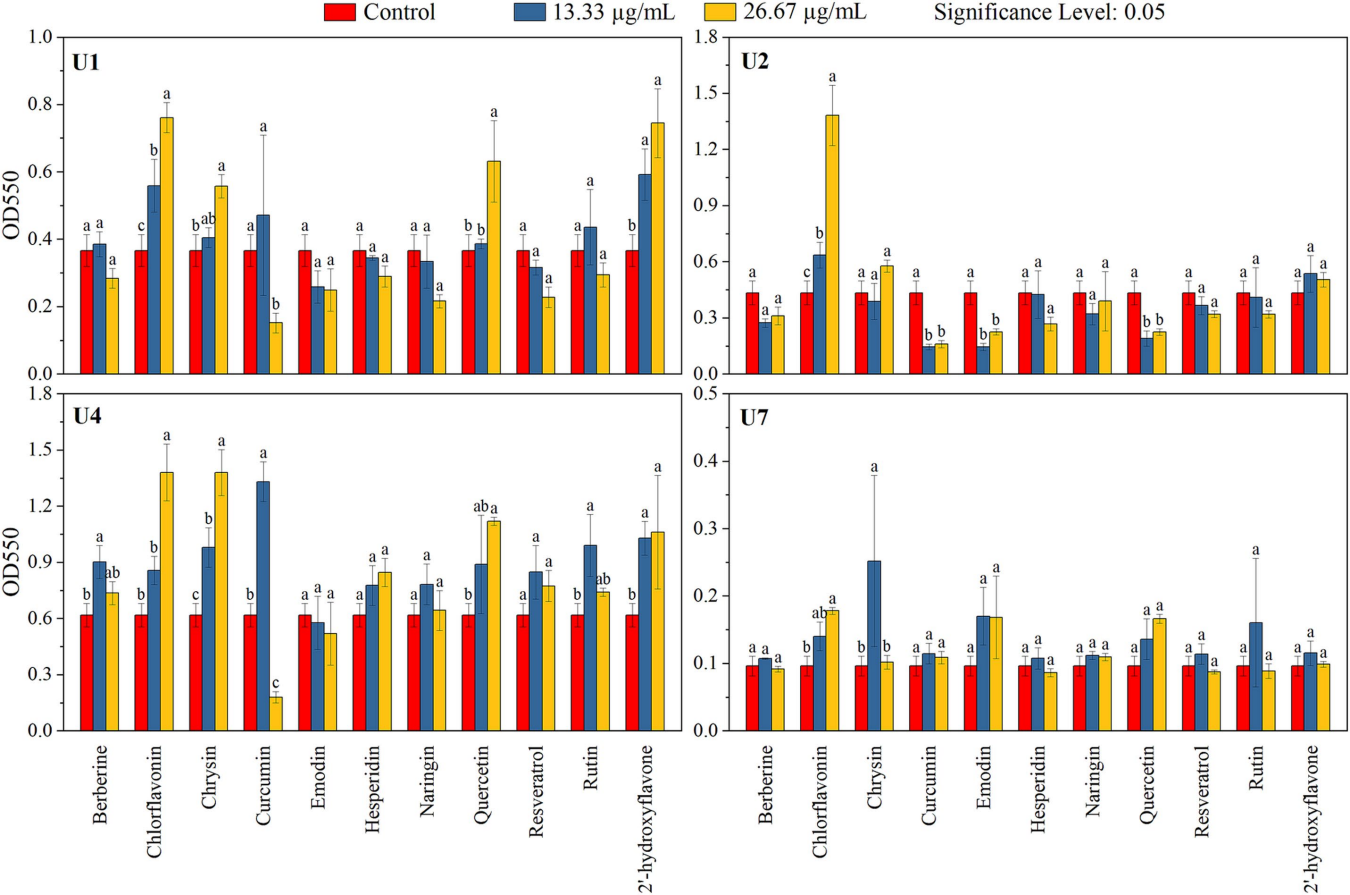
Effects of 11 natural compounds on biofilm formation of multiple-antibiotic-resistant bacteria (i.e., U1, U2, U4, and U7). Differences between treatment groups were analyzed using paired comparison plots. Different letters indicate statistically significant differences at the *p* < 0.05 level.

In parallel, the inhibitory effects of the 11 compounds on biofilm formation were assessed (Figure 4; Table 2). For *Microbacterium* sp. U1, only curcumin at 26.67 μg/mL significantly inhibit the biofilm formation (*p* < 0.05). In contrast, chlorflavonin and 2′-hydroxyflavone at both concentrations, as well as chrysin and quercetin at 26.67 μg/mL, significantly promoted the biofilm formation. For *Microbacterium* sp. U2, curcumin, emodin, and quercetin inhibited the biofilm formation significantly at both concentrations (*p* < 0.05), while chlorflavonin significantly promoted it. For *Lactococcus lactis* sp. U4, curcumin showed dual behavior: significant inhibition at 26.67 μg/mL but promotion at 13.33 μg/mL (*p* < 0.05). Biofilm promotion was also observed with 13.33 μg/mL berberine, 26.67 μg/mL chlorflavonin, both concentrations of chrysin, 26.67 μg/mL quercetin and 13.33 μg/mL rutin. For *Chryseobacterium* sp. U7, no compound significantly inhibited biofilm formation. However, chlorflavonin at 26.67 μg/mL and chrysin at 13.33 μg/mL significantly enhanced biofilm formation (*p* < 0.05). In summary, curcumin and emodin demonstrated the strongest anti-biofilm activity among the tested compounds, particularly against Gram-positive strains. *Chryseobacterium* sp. U7 exhibited a high tolerance to all compounds in terms of both growth and biofilm formation, highlighting the challenge of controlling Gram-negative antibiotic-resistant bacteria with natural compounds. This trend was further supported by results from *Pseudomonas aeruginosa* PAO1, another Gram-negative species, whose biofilm formation remained unaffected by any tested compounds (Supplementary Figure S4).

### 3.5 Effect of selected natural compounds on motility of multiple-antibiotic-resistant bacteria

**Figure 5:**
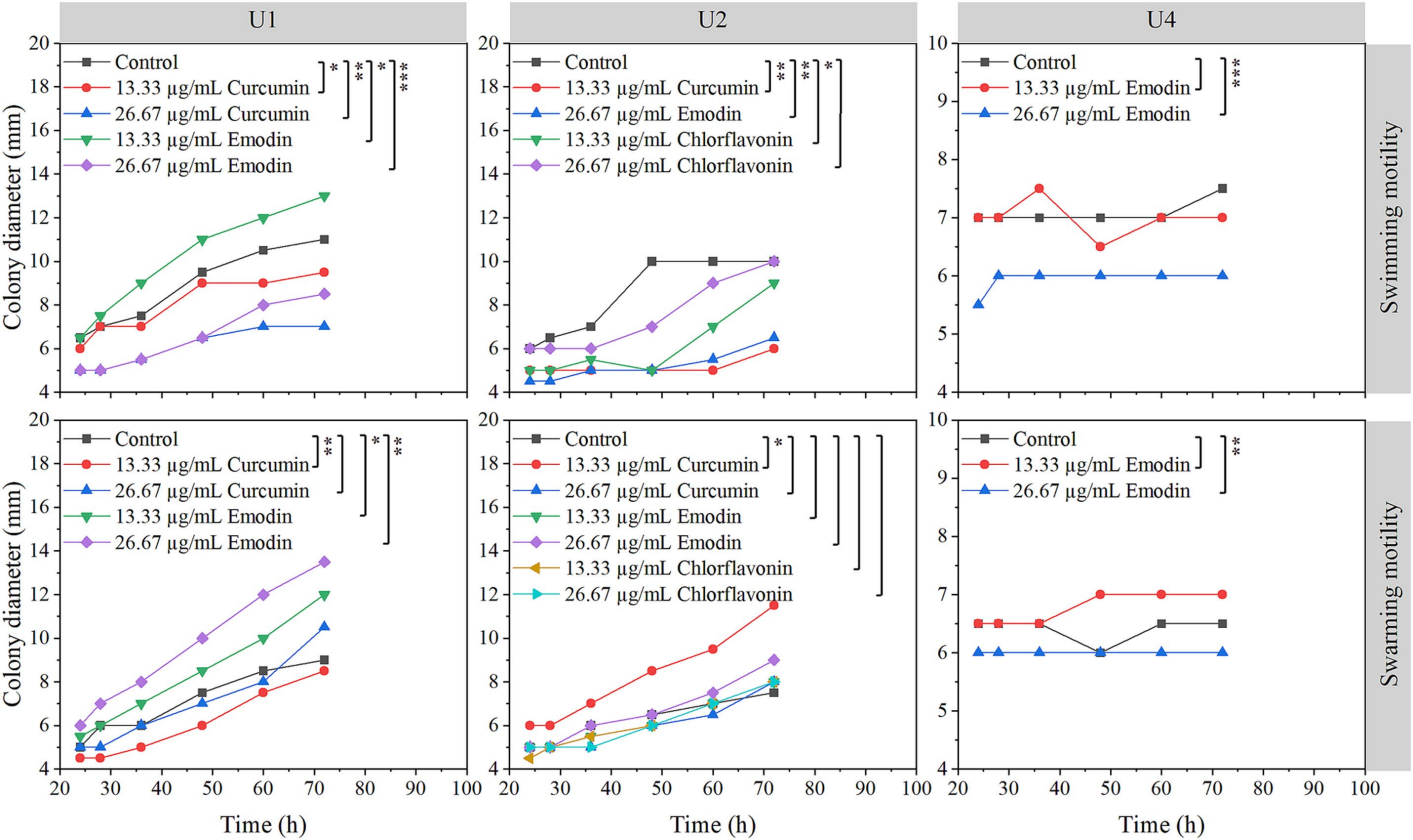
Effects of selected natural compounds on motility of multiple-antibiotic-resistant bacteria (i.e., U1, U2, and U4). The variation between different treatments was calculated by a paired- samples *t*-test; *, *p* < 0.05; **, *p* < 0.01; ***, *p* < 0.001.

The effects of selected natural compounds on bacterial motility were assessed for three strains: *Microbacterium* sp. U1 (treated with curcumin and emodin), *Microbacterium* sp. U2 (treated with curcumin, emodin, and chlorflavonin), and *Lactococcus lactis* sp. U4 (treated with emodin) (Figure 5). For *Microbacterium* sp. U1, swimming motility was significantly inhibited by both concentrations of curcumin and by 26.67 μg/mL emodin (*p* < 0.05), whereas 13.33 μg/mL emodin significantly promoted swimming motility, as evidenced by increased colony diameter. In contrast, swarming motility was only significantly inhibited by 13.33 μg/mL curcumin, while both concentrations of emodin significantly enhanced swarming motility (*p* < 0.05). These results suggested that 13.33 μg/mL curcumin effectively reduces both swimming and swarming motility in U1. For *Microbacterium* sp. U2, swimming motility was significantly inhibited by 13.33 μg/mL curcumin, 26.67 μg/mL emodin, and 13.33 μg/mL chlorflavonin (*p* < 0.05). However, no significant differences were observed in swarming motility across treatments, indicating a greater resistance of swarming behavior to compound exposure. For *Lactococcus lactis* sp. U4, 26.67 μg/mL emodin significantly inhibited both swimming and swarming motility (*p* < 0.01), highlighting its potential as an effective motility-suppressing agent in this strain.

## 4 Discussion

### 4.1 Profiling of antibiotic-resistant bacteria (ARB) in WWTPs

The bacteria isolated in this study were *Microbacterium* spp., *Chryseobacterium* spp., *Lactococcus lactis* spp., and *Psychrobacter* spp., which may not be commonly detected or considered representative ARB in WWTPs (Rizzo et al., 2013). However, all four genera have been widely reported to display antibiotic resistance. For example, 13 of 16 isolated *Microbacterium* spp. from contaminated soil showed tolerance to ampicillin, chloramphenicol, and vancomycin (Learman et al., 2019). Four multidrug-resistant *Chryseobacterium* strains were isolated from activated sludge collected at domestic wastewater treatment facilities (Pham and Li, 2024). *Lactococcus lactis* strains exhibited natural resistance to antibiotics and possessed phenotypic resistance to most of the twenty tested antibiotics (Hamdaoui et al., 2024). Analysis of genome sequences showed that genes related to antibiotic resistance were identified in three *Psychrobacter* sp. strains (Lasa and Romalde, 2017). These bacteria originated from different environments and preferred different conditions. *Microbacterium* spp. are environmentally present bacteria that require complex nutrients (Yu et al., 2023). Members of the genus *Chryseobacterium* are among such human pathogens that cause a myriad of nosocomial infections, including pneumonia, bacteremia, biliary tract, and intra-abdominal infections (Kirby et al., 2004). *Lactococcus lactis* spp. are the main ingredient of numerous industrial and starter cultures. *Psychrobacter* spp. are more frequently found in cold and other non-polar environments with low water activity (Rodrigues et al., 2009). Their detection in treated effluent raises concerns about the potential release of uncommon but resilient ARBs into receiving environments, where they may transfer resistance genes to native microbial communities or opportunistic pathogens.

While these uncommon ARBs may not currently be prioritized by global health organizations, their potential to act as ARG reservoirs cannot be overlooked. Regarding their resistance to the selected antimicrobials, the WHO has categorized 12 families of bacteria that pose the utmost threat to human and environmental health into three classes: medium, high, and critical (Supplementary Table S2) (Mojia, 2017). The critical group includes multidrug-resistant bacteria that are very fatal and mostly lethal infections. They include *Acinetobacter baumannii*, *Pseudomonas aeruginosa*, and Enterobacteriaceae (including *Escherichia coli*, *Klebsiella* spp., *Proteus* spp., and *Serratia* spp.). Common infection-causing bacteria form the high priority group, like *Enterococcus faecium*, *Staphylococcus aureus*, *Helicobacter pylori*, *Salmonellae* spp., and *Neisseria gonorrhoeae*. The medium group includes *Streptococcus pneumoniae*, *Haemophilus influenzae*, and *Shigella* spp. Other bacteria (such as *Tuberculosis* and *streptococcus* A and B) not included in this list also showed resistance to antibiotics. In WWTPs, many bacteria in the list have been detected or isolated, such as Enterobacteriaceae (*Escherichia coli* and *Klebsiella pneumoniae*), *Staphylococcus aureus*, *Enterococcus faecium*, *Acinetobacter baumannii, Pseudomonas aeruginosa*, and *Campylobacter* spp. (Galler et al., 2018; Jałowiecki et al., 2022). Additionally, *Aeromonas* spp., *Enterococci, Acinetobacter* spp., and *Pseudomonas* spp. were also observed in WWTPs (Bouki et al., 2013; Galler et al., 2018; Li et al., 2022). Rizzo et al. (2013) reviewed that *Enterococci*, *E. coli*, and *Acinetobacter* spp. are among the most widely investigated bacteria for assessing the spread of AR in WWTPs. This highlights the need to expand monitoring beyond typical fecal indicators to include environmental bacteria such as *Microbacterium* spp. that may act as hidden reservoirs of resistance genes.

### 4.2 Antibiotic resistance of the isolated antibiotic-resistance bacteria from WWTP effluent

In this study, *Microbacterium* spp. showed resistance to the largest number of antibiotics, followed by *Chryseobacterium* spp., *Lactococcus lactis* spp., and *Psychrobacter* sp. *Microbacterium* sp. U2 was resistant to all 8 tested antibiotics, and *Microbacterium* sp. U1 showed tolerance to 6 of 8 tested antibiotics (Table 3). According to the results of BLAST, U1 and U2 were identified as *Microbacterium oxydans* and *Microbacterium mantypicum* (Supplementary Table S1; Figure 1B). *Microbacterium oxydans* was reported resistant to ampicillin, kanamycin, chloramphenicol and streptomycin at high levels, while there are no publications about the antibiotic resistance of *Microbacterium mantypicum* (Ozaktas et al., 2012) (Table 3). *Microbacterium* isolates have been previously documented to tolerate various antibiotics, such as *β*-lactams (ampicillin), aminoglycosides (kanamycin), macrolides (erythromycin), quinolones (norfloxacin, ciprofloxacin), tetracyclines (tetracycline), sulfonamides (sulfamethazine, sulfamethoxazole, sulfadiazine, or sulfadimethoxine), chloramphenicol and vancomycin (Kim et al., 2011; Luthra et al., 2018; Learman et al., 2019; Billet et al., 2021; Sodhi et al., 2021). It has been noted that *Mycobacterium tuberculosis* has intrinsic resistance to earlier β-lactams and is resistant to virtually all other antibiotics (Fisher and Mobashery, 2016). These findings suggest that *Microbacterium* strains are important multiple-antibiotic-resistant bacteria. Their broad resistance profiles indicate significant potential to act as reservoirs of ARGs, especially in wastewater environments. The broad resistance profiles observed in *Microbacterium* strains may stem from both environmental adaptation and potential exposure to anthropogenic antibiotics in WWTP influent. Given their previously reported ability to degrade or tolerate sulfonamides and β-lactams (Billet et al., 2021), these strains may acquire resistance through both selection and horizontal gene transfer. The detection of multiple efflux pumps and β-lactamase genes (e.g., *ampC*) further supports the likelihood of acquired resistance rather than intrinsic tolerance alone.

**Table 3:** The comparison of antibiotic resistance of the isolated strains in this study and previous studies (mentioned in the text).

Antibiotic (Classification)	*Microbacterium*			*Chryseobacterium*				*Lactococcus lactis*				*Psychrobacter*	
	U1	U2	*Oxydans*	U3	U5	U7	*Aquaticum*	U4	U6	U8	*Subsp. lactis*	U9	*Pulmonis*
Sulfamethoxazole (Sulfonamides)	+	+		+	+	+	+	+	+	+	+	+	
Carbenicillin (β-Lactams)	+	+		+	+	+		−	−	−		−	
Chloramphenicol (Amphenicols)	−	+	+	−	+	+		−	−	−	−	−	
Erythromycin (Macrolides)	+	+		−	−	+		−	−	−		−	
Kanamycin (Aminoglycosides)	+	+	+	+	+	+		−	−	−		−	+
Nalidixic acid (Quinolones)	+	+		−	−	−		+	+	+		−	
Tetracycline (Tetracyclines)	−	+		−	−	−		−	−	−	−	−	−
Colistin (Polymyxins)	+	+		+	+	+		+	+	+		+	
Ampicillin (β-Lactams)			+								−		
Streptomycin (Aminoglycosides)			+								+		
Florfenicol (Amphenicols)							+						
Enrofloxacin (Quinolones)							+						
Oxytetracycline (Tetracyclines)							+						
Amoxicillin (β-Lactams)							+				−		
Trimethoprim (Folate antagonists)							+				+		
Meropenem (β-Lactams)											+		
Cefotaxime (β-Lactams)											+		
Ceftriaxone (β-Lactams)											+		
Cefepime (β-Lactams)											+		
Imipenem (β-Lactams)											+		
Ertapenem (β-Lactams)											+		
Clindamycin (Lincosamides)											+		
Penicillin G (β-Lactams)											−		−
Teicoplanin (Glycopeptides)											−		
Vancomycin (Glycopeptides)											−		
Gentamicin (Aminoglycosides)											−		−
Rifampicin (Ansamycins)											−		

(+), resistance; (−), no resistance; (blank), no available data was found.

*Chryseobacterium* spp. U3, U5 and U7 showed tolerance to four, five and six of eight tested antibiotics, respectively (Table 3). U3 and U5 have high similarity with *Chryseobacterium* sp. HP3E and *Chryseobacterium* sp. PDD-58b-7, while U7 have high similarity with *Chryseobacterium* sp. 5,127 and *Chryseobacterium aquaticum strain* KR2-2 (Supplementary Table S1; Figure 1B). *Chryseobacterium* spp., environmental bacteria, which are primarily found in soil and water, have increasingly been found to colonize immunocompromised patients through contaminated medical devices and liquids (Esposito et al., 2015). It was reported that most of the *Chryseobacterium aquaticum* strains isolated from farmed salmonids could grow even in the presence of high concentrations of the tested antimicrobials (florfenicol, enrofloxacin, oxytetracycline, amoxicillin, sulfamethoxazole and trimetoprim) (Saticioglu et al., 2021) (Table 3). Isolates of *Chryseobacterium* spp. were among the most antibiotic-resistant bacteria selected in agricultural soils (Armalytė et al., 2019), suggesting they are non-negligible antibiotic-resistant bacteria. Their occurrence in both clinical and environmental settings underscores their adaptability and resistance potential.

*Lactococcus lactis* spp. U4, U6 and U8 all showed tolerance to three of the eight tested antibiotics, namely sulfamethoxazole, nalidixic acid and colistin (Table 3). U4 showed high similarity with *Lactococcus lactis sub*sp. *lactis* IO-1 and *Lactococcus lactis subsp. lactis strain* A12; U6 showed high similarity with *Lactococcus lactis strain* HBUAS58280 and *Lactococcus lactis strain* 4,355; and U8 showed high similarity with *Lactococcus lactis strain* HBUAS58280 and *Lactococcus lactis strain* Mise173 (Supplementary Table S1; Figure 1B). For *Lactococcus lactis subsp. lactis*, antibiotic resistance testing showed resistance to meropenem, cefotaxime, ceftriaxone, cefepime, imipenem, ertapenem, clindamycin, sulfamethoxazole, trimethoprim and streptomycin, while sensitive was observed to ampicillin, amoxicillin, penicillin G, teicoplanin, vancomycin, gentamicin, rifampicin, tetracycline and chloramphenicol (Hamdaoui et al., 2024) (Table 3). This result is consistent with our study. *Lactococcus lactis* spp. isolates can be susceptible to some antibiotics but resistant to others, in accordance with profiles reported for the subspecies (Khemariya et al., 2017). Importantly, *Lactococcus lactis* is also known to carry resistance genes on mobile genetic elements such as plasmids and transposons, increasing its potential for horizontal gene transfer in microbial communities. Therefore, *Lactococcus lactis* spp. must be carefully characterized to ensure the absence of acquired antimicrobial resistance, particularly when used as probiotic cultures for food applications (Khemariya et al., 2017).

*Psychrobacter* sp. U9 only showed resistance to two of eight tested antibiotics, namely sulfamethoxazole and colistin (Table 3). U9 was identified as *Psychrobacter pulmonis strain* MB36 and *Psychrobacter* sp. BSw20884b (Supplementary Table S1; Figure 1B). An isolate with 88.54% sequence similarity to *Psychrobacter pulmonis* from soil was sensitive to gentamicin, penicillin, and tetracycline but resistant to kanamycin (Hawas et al., 2024) (Table 3). In general, *Psychrobacter* spp. strains exhibit high susceptibility to various antimicrobial drugs. Data from patient isolates showed that they were mostly susceptible to *β*-lactam antibiotics (cell-wall construction inhibitors), protein synthesis inhibitors (macrolides, aminoglycosides and tetracyclines) and DNA coiling (quinolones) (Caspar et al., 2013; Ioannou et al., 2025). Resistance to vancomycin, penicillin, aminopenicillins, and macrolides was observed in only a few cases (Ioannou et al., 2025). Nevertheless, recent metagenomic analyses suggest that *Psychrobacter* might serve as ecological reservoirs for the transfer of various resistance gene markers to other microbes (Cuadrat et al., 2020). Consequently, knowledge on antibiotic resistance in the genus *Psychrobacter* is largely lacking, and further investigations of sensitivity assays remain crucial.

### 4.3 Molecular mechanisms of antibiotic resistance of isolated antibiotic-resistance bacteria

To better understand the resistance patterns observed in WWTP effluent, we investigated the underlying genetic mechanisms of antibiotic resistance in the isolated strains through whole genome sequencing. In general, there are two types of antibiotic resistance mechanisms, i.e., intrinsic-resistance and acquired-resistance. Intrinsic-resistance refers to inherent structural or functional characteristics and usually exists in antibiotic-producing bacteria. Acquired resistance results from horizontal gene transfer (HGT) or mutation in chromosomal genes. These mechanisms can be categorized into four main groups (Blair et al., 2015; Soni et al., 2022; Darby et al., 2023): (i) mechanisms that minimize the intracellular antibiotic concentrations, such as reduced permeability of antibiotics across the cell membrane and increased efflux that bacterial efflux pumps actively transport many antibiotics out of the cell; (ii) mechanisms that act on antibiotic targets, such as modification or alteration of the target by genetic mutation or post-translational modification, and target protection that proteins bind to antibiotic targets to prevent the antibiotic molecules from attaining their binding site; (iii) mechanisms that modify or destroy the antibiotic itself, such as antibiotic inactivation by hydrolysis or modification by the transfer of a chemical group; (iv) mechanisms that bacteria circumvent inhibited pathways, i.e., bypass of the metabolic pathways. In addition to these, biofilm formation plays a crucial role in antibiotic resistance by creating a protective microenvironment and limiting antibiotic penetration (Stewart, 2002; Balcázar et al., 2015).

Whole genome sequencing revealed that *Microbacterium* spp. U1 and U2, *Lactococcus lactis* sp. U4, and *Chryseobacterium* sp. U7 contained genes conferring resistance to aminoglycoside, *β*-lactam, chloramphenicol, quinolones, sulfonamides, and tetracyclines. *Lactococcus lactis* sp. U4 and *Chryseobacterium* sp. U7 also harbored genes for streptogramins resistance (Figure 2A). These findings are consistent with the antibiotic susceptibility profiles, confirming that these isolates exhibit multidrug resistance. Among sulfonamide resistance genes, *sul1*, *sul2*, *sul3*, and *sulA* are the most commonly documented, coding for sulfonamide-insensitive dihydropteroate synthase isoproteins (Yun et al., 2012; Billet et al., 2021). Our study showed that U1, U2, U4 and U7 had *sul3* gene and displayed resistance to sulfamethoxazole (Table 3; Figure 2A). The consistent resistance to sulfamethoxazole across all isolates suggests a strong selective pressure from sulfonamide exposure, which is commonly used in both human and veterinary medicine. *sul3* is a known marker of sulfamethoxazole resistance and is commonly associated with anthropogenic sources, including hospital and domestic wastewater (Chen et al., 2023). In general, *sul1* and *sul2* genes are more frequently found in environmental samples, while *sul3* gene was mainly associated with animal- or human-derived sources (Felis et al., 2024). Environmental studies typically report *sul3* at lower frequencies than *sul1* and *sul2* (Phuong Hoa et al., 2008). Interestingly, in our urban WWTP effluent samples, only *sul3* was detected, and *sul1* and *sul2* genes were absent, suggesting a predominantly human-derived ARG profile in the effluent.

U1, U2, and U4 are Gram-positive bacteria, while U7 is Gram-negative bacteria. The *β*-lactam resistance genes in U7 are quite different from those in U1, U2, and U4 (Figure 2A). U1 and U2 carried *ampC*, related to resistance via *β*-lactamase degradation and altered penicillin-binding proteins. And U2 also harbored *IreK* and *amlv*, which contributes to *β*-lactam resistance through regulation of cell wall stress responses. Antibiotic degradation by *β*-lactamase and alteration in penicillin-binding membrane proteins are the main mechanisms of Gram-positive pathogens resistant to *β*-lactam antibiotics (Kernodle, 2006). For example, AmpC *β*-lactamases played vital roles in *β*-lactams resistance (Hussain et al., 2021). The *ampC* gene was detected in *Microbacterium* spp. U1 and U2, which can explain their resistance to carbenicillin (Table 3). However, *ampC* gene and resistance to carbenicillin were not observed in *Lactococcus lactis* sp. U4. In Gram-negative bacteria, most *β*-lactams were hydrolyzed by highly transferable plasmid-mediated *β*-lactamases (Hussain et al., 2021). In contrast, U7 lacked the *ampC* gene but contained multiple *β*-lactamase resistance genes (8 types), including *ADC-129*, *CMA*, *CME*, *CMY*, *CPS*, *EPS* and *PEDO-2*, which likely contributed to its carbenicillin tolerance (Table 3). These genes are commonly plasmid-mediated and prevalent in Gram-negative bacteria.

Tetracycline resistance generally arises from the newly acquisition genes, which were most identified as *tet* genes (Roberts and Schwarz, 2017). *tet*(T) encodes a ribosomal protection protein, while *tet*(X) encodes an enzyme that inactivates tetracycline (Roberts and Schwarz, 2017). Although *tet*(T) and *tet*(X) were found in U1, U2, U4 and U7 via whole genome sequencing, only *Microbacterium* sp. U2 exhibited phenotypic resistance (Table 3; Figure 2A). This discrepancy suggests possible gene silencing or insufficient expression under the tested conditions. Chloramphenicol resistance primarily results from either efflux pumps (e.g., *cmlv*) or inactivation by chloramphenicol acetyltransferases (e.g., *catB3*) (Roberts and Schwarz, 2017). In our study, *cmlv* was detected in U1, U2, and U4, while *catB3* was found in U4 and U7 (Figure 2A). U2 and U7 were resistant to chloramphenicol, consistent with the presence of *cmlv* and *catB3*, respectively (Table 3). However, although U1 harbored *cmlv* and *Lactococcus lactis* sp. U4 harbored *cmlv* and *catB3*, they did not show tolerance to chloramphenicol.

Aminoglycoside resistance mechanisms include enzymes inactivation, mutations or modifications of the ribosome target, reduced permeability, and efflux pumps (Garneau-Tsodikova and Labby, 2016). For example, AAC(6′)-lz, AAC(3)-lb/AAC(6′), APH(3′), aminoglycoside-modifying enzymes, can inactivate aminoglycosides, including kanamycin (Garneau-Tsodikova and Labby, 2016; Zárate et al., 2019). Genes encoding aminoglycoside-modifying enzymes were observed in U1, U2, and U4 (Figure 2A). Despite this, only U1, U2, and U7 showed resistance to kanamycin (Table 3). This further illustrates that gene presence does not always correlate with phenotypic resistance.

In addition to *β*-lactam and other antibiotic classes, we investigated quinolone resistance in the isolated strains. Bacteria generally resist quinolones through three main mechanisms (Solano-Gálvez et al., 2020): (i) chromosomal mutations in target genes that reduce the binding affinity of quinolones (e.g., mutations in *gyrA*, *gyrB*, or *parC*); (ii) mutations or changes that reduct the intracellular concentration of quinolones, such as through efflux pumps or decreased membrane permeability; and (iii) plasmid-mediated quinolone resistance genes (e.g., *qnr* genes) that project target enzymes. In our study, *gyrA* and *gyrB* were annotated for *Microbacterium* sp. U1; *gyrA*, *gyrB*, and *QnrC* were identified in *Microbacterium* sp. U2; *gyrA*, *gyrB*, *QnrC*, and *parC* were annotated for *Lactococcus lactis* sp. U4; and *gyrB*, *QnrC*, and *parC* were annotated for *Chryseobacterium* sp. U7 based on whole genome sequencing (Figure 2A). Genes *gyrA*, *gyrB*, and *parC* were involved in chromosomal mutation-based resistance, while *qnrC* and *parC* can play roles in plasmid-mediated protection (Solano-Gálvez et al., 2020). Antibiotic sensitivity tests showed that U1, U2, and U4 had resistance to nalidixic acid, but U7 was not (Table 3). Previous studies have shown that quinolone resistance in Gram-negative bacteria is commonly associated with mutations in *gyrA* (Pan and Fisher, 1997), which was absent in U7, potentially explaining its susceptibility.

Beyond resistance to individual antibiotic classes, all four strains (U1, U2, U4, and U7) also carried genes associated with MDR. These include genes for ABC efflux pump, MFS efflux pump, ABC-F ribosomal protection proteins, penicillin-binding protein mutations, RND efflux pump, SMR efflux pump, colistin resistance transmembrane protein and so on (Figure 2A). Notably, only *Chryseobacterium* sp. U7 harbored the colistin resistance transmembrane protein; yet phenotypically, all four strains exhibited resistance to colistin (Table 3). Colistin resistance, observed even in non-clinical genera such as *Microbacterium* and *Lactococcus*, raises concerns due to colistin’s role as a last-resort antibiotic. While plasmid-mediated *mcr* (mobilized colistin resistance) genes were not detected, the presence of efflux pump and membrane protein genes related to polymyxin resistance in Gram-negative *Chryseobacterium* may suggest chromosomal or intrinsic mechanisms. Further, the detection of colistin resistance in *Psychrobacter*, a cold-adapted genus, suggests that resistance traits may be more widespread in environmental bacteria than previously assumed. These findings highlight a common observation in antimicrobial resistance studies: the presence of resistance genes (ARGs) does not always directly correlate with observed phenotypic resistance. Such discrepancies may result from silent gene expression, lack of induction under test conditions, or environmental stress factors that influence gene activity (Andersson and Hughes, 2010; Partridge et al., 2018).

### 4.4 Antibacterial mechanisms of natural compounds against multiple-antibiotic-resistant bacteria

Despite growing interest in natural antimicrobials, studies specifically evaluating their effects on antibiotic-resistant bacteria from urban wastewater remain scarce. Antibiotic resistance is a global health crisis, necessitating the development of novel antimicrobial agents and strategies with reduced propensity for resistance development. In this study, several natural compounds were screened for their antibacterial effects on multiple-antibiotic-resistant isolates. Emodin inhibited the growth of *Microbacterium* spp. U1 and U2, as well as *Lactococcus lactis* sp. U4. Similarly, curcumin limited the cell growth of U1 and U2, while quercetin affected U4, and chloflavonin inhibited the growth of U2. Moreover, curcumin suppressed biofilm formation of U1, U2, and U4, whereas emodin and quercetin inhibited the biofilm formation of U2. Curcumin and emodin also reduced the motility of U1 and U2. Collectively, curcumin and emodin demonstrated the most consistent and broad-spectrum antibacterial and antibiofilm activities across multiple strains, followed by quercetin and chlorflavonin. Their differential effectiveness against various phenotypes suggests diverse and complementary mechanisms of action.

Natural products are increasingly recognized as promising alternatives or adjuncts to conventional antibiotics due to their structural diversity and multi-target activity. They can inhibit bacterial growth or virulence through various mechanisms, including membrane disruption, interference with DNA/RNA synthesis, quorum sensing inhibition, efflux pump suppression, and biofilm inhibition (Lee et al., 2016; Xiu et al., 2017; Arrigoni et al., 2024; Santacroce et al., 2024). Various natural compounds were described for their ability to prevent biofilm formation through different approaches (Arrigoni et al., 2024). Such multi-targeted activity may mitigate resistance development and potentiate synergistic effects when used in combination with traditional antibiotics.

Curcumin, a plant-derived polyphenolic compound, exhibits broad-spectrum antibacterial properties and strong anti-inflammatory activity. It inhibits cell growth by affecting the cell wall, cell membrane, protein, DNA, or other cellular structures (Zheng et al., 2020), which can explain its limitation to cell growth of U1 and U2 isolated in this study. Furthermore, curcumin significantly inhibited biofilm formation in U1, U2, and U4. Biofilm development involves a multi-step process: initial attachment, microcolony formation, maturation, and dispersion (Sauer et al., 2022). Quorum sensing, a bacterial communication system, plays a critical role in regulating genes involved in biofilm maturation and virulence. Curcumin disrupts this communication, thereby preventing biofilm formation rather than eradicating established biofilms (Zheng et al., 2020). This mechanism explains its effectiveness in early-stage biofilm inhibition without necessarily reducing viability. Additionally, curcumin has shown potential for combination therapy, where it exhibits additive or synergistic effects with conventional antibiotics, potentially enhancing treatment outcomes and minimizing resistance emergence (Zheng et al., 2020).

Emodin, an anthraquinone derived from traditional medicinal plants, also demonstrated strong antibacterial effects. It exerts its action by penetrating phospholipid bilayers, altering membrane fluidity, and increasing cell membrane permeability, leading to cellular leakage and structural disruption (Lee et al., 2016). For example, Li et al. (2016) reported that emodin altered the membrane structure of *Haemophilus parasuis*, thereby enhancing antibiotic uptake. In our study, emodin emerged as the most effective natural product in limiting the growth of multiple strains, particularly *Microbacterium* sp. U2. Moreover, emodin inhibited biofilm formation in U2, possibly by disrupting extracellular polymeric substances (EPS) or interfering with biofilm matrix proteins. Đukanović et al. (2022) demonstrated emodin’s potential as a novel antibiofilm agent, showing inhibition of polysaccharide intercellular adhesin production, a key component in *Staphylococcus aureus* biofilm formation. These findings support the role of emodin as a potential anti-virulence agent targeting bacterial communication and surface adhesion.

Quercetin, another plant-derived polyphenolic compound, possesses antimicrobial activity and anti-inflammatory function. It can damage bacterial membranes, interfere with nucleic acid synthesis, and suppress protein expression. In addition, quercetin has been reported to inhibit virulence factors, disrupt energy metabolism, and attenuate quorum sensing (Nguyen and Bhattacharya, 2022; Arrigoni et al., 2024). In our study, quercetin inhibited the growth of *Lactococcus lactis* sp. U4 and biofilm of *Microbacterium* sp. U2. A recent study further confirmed that quercetin inhibited the formation of biofilms and reduced the expression of genes linked to biofilm production as well as ARGs in wastewater *Aeromonas* (Judan Cruz et al., 2024). The dual activity of quercetin against both planktonic cells and biofilms underscores its versatility as an antimicrobial agent. Chlorflavonin, a less commonly studied flavonoid, showed inhibitory activity on the growth of *Microbacterium* sp. U2, although no antibiofilm effect was observed. This may reflect its primary bacteriostatic or bactericidal mode of action rather than anti-virulence activity. Previous research has documented antibacterial activity of clorflavonin against *Mycobacterium tuberculosis*, suggesting its potential against targeting slow-growing or intracellular pathogens.

Notably, none of the 11 natural compounds exerted significant effects on cell growth and biofilm formation of Gram-negative bacteria *Chryseobacterium* sp. U7 (Table 2). Comparable results were observed for *Pseudomonas aeruginosa* PAO1, a model Gram-negative bacterium (Supplementary Figure S4). This resistance may be attributed to the unique structural characteristics of Gram-negative bacteria, including their outer membrane barrier, low membrane permeability, and expression of efflux pumps, which collectively hinder compound uptake and retention. Although previous studies have reported that curcumin and quercetin could be against both Gram-positive and Gram-negative bacteria (Tyagi et al., 2015; Nguyen and Bhattacharya, 2022), our findings suggest that compound efficacy may be species-specific and dependent on strain-level variations in membrane composition or resistance mechanisms. These observations underscore the need for further research into how bacterial cell envelope structures influence natural compound activity, and how such compounds can be optimized, through formulation or structural modification, to enhance efficacy against Gram-negative pathogens.

## 5 Conclusion

In the present study, nine antibiotic-resistant bacteria were isolated from the WWTP effluent, including *Microbacterium* spp. (Actinobacteria), *Chryseobacterium* spp. (Bacteroidetes), *Lactococcus lactis* spp. (Firmicutes), and *Psychrobacter* sp. (Proteobacteria), which are not typically considered as predominant or representative ARB in WWTPs. Whole-genome sequencing and annotation revealed that these multiple-antibiotic-resistant isolates harbored a diverse array of ARGs, consistent with their observed resistance to multiple antimicrobial classes. Furthermore, eleven natural compounds were tested for their effects on cell growth, biofilm formation, and motility of these multiple-antibiotic-resistant isolates. Among them, curcumin and emodin showed consistent and broad-spectrum activity, significantly inhibiting both growth and biofilm formation in several resistant strains. Quercetin and chlorflavonin also showed selective antibacterial effects, particularly against *Lactococcus* and *Microbacterium* isolates. Our work applies whole-genome sequencing to cultured, multidrug-resistant environmental isolates, enabling high-resolution insights into ARG distribution and potential HGT mechanism. In addition, we are among the first to screen a panel of natural compounds against these isolates and evaluate their impacts on bacterial growth, motility, and biofilm formation. By linking genotypic profiles to functional antimicrobial responses, our study establishes a novel framework for assessing environmentally relevant strategies to mitigate the spread of antibiotic resistance in wastewater systems.

## Data Availability

The datasets presented in this study can be found in online repositories. The names of the repository/repositories and accession number(s) can be found in the article/Supplementary material.

## References

[ref1] Alam K. Al Farraj D. A. Mah-e-Fatima S. Yameen M. A. Elshikh M. S. Alkufeidy R. M.. (2020). Anti-biofilm activity of plant derived extracts against infectious pathogen-*Pseudomonas aeruginosa* PAO1. J. Infect. Public Health 13, 1734–1741. doi: 10.1016/j.jiph.2020.07.007 32753311

[ref2] Al-Mustapha A. I. Tiwari A. Laukkanen-Ninios R. Lehto K.-M. Oikarinen S. Lipponen A.. (2025). Wastewater based genomic surveillance key to population level monitoring of AmpC/ESBL producing *Escherichia coli*. Sci. Rep. 15:7400. doi: 10.1038/s41598-025-91516-9, PMID: 40033002 PMC11876440

[ref3] AlSheikh H. M. A. Sultan I. Kumar V. Rather I. A. Al-Sheikh H. Tasleem Jan A.. (2020). Plant-based phytochemicals as possible alternative to antibiotics in combating bacterial drug resistance. Antibiotics 9:480. doi: 10.3390/antibiotics9080480, PMID: 32759771 PMC7460449

[ref4] Andersson D. I. Hughes D. (2010). Antibiotic resistance and its cost: is it possible to reverse resistance? Nat. Rev. Microbiol. 8, 260–271. doi: 10.1038/nrmicro2319 20208551

[ref5] Armalytė J. Skerniškytė J. Bakienė E. Krasauskas R. Šiugždinienė R. Kareivienė V.. (2019). Microbial diversity and antimicrobial resistance profile in microbiota from soils of conventional and organic farming systems. Front. Microbiol. 10:892. doi: 10.3389/fmicb.2019.00892, PMID: 31105678 PMC6498881

[ref6] Arrigoni R. Ballini A. Jirillo E. Santacroce L. (2024). Current view on major natural compounds endowed with antibacterial and antiviral effects. Antibiotics 13:603. doi: 10.3390/antibiotics13070603, PMID: 39061285 PMC11274329

[ref7] Balcázar J. L. Subirats J. Borrego C. M. (2015). The role of biofilms as environmental reservoirs of antibiotic resistance. Front. Microbiol. 6:1216. doi: 10.3389/fmicb.2015.01216, PMID: 26583011 PMC4628128

[ref8] Billet L. Pesce S. Rouard N. Spor A. Paris L. Leremboure M.. (2021). Antibiotrophy: key function for antibiotic-resistant bacteria to colonize soils — case of sulfamethazine-degrading *Microbacterium* sp. C448. Front. Microbiol. 12:643087. doi: 10.3389/fmicb.2021.643087, PMID: 33841365 PMC8032547

[ref9] Blair J. M. A. Webber M. A. Baylay A. J. Ogbolu D. O. Piddock L. J. V. (2015). Molecular mechanisms of antibiotic resistance. Nat. Rev. Microbiol. 13, 42–51. doi: 10.1038/nrmicro3380, PMID: 25435309

[ref10] Bouki C. Venieri D. Diamadopoulos E. (2013). Detection and fate of antibiotic resistant bacteria in wastewater treatment plants: a review. Ecotoxicol. Environ. Saf. 91, 1–9. doi: 10.1016/j.ecoenv.2013.01.016, PMID: 23414720

[ref11] Brlek P. Bulić L. Bračić M. Projić P. Škaro V. Shah N.. (2024). Implementing whole genome sequencing (WGS) in clinical practice: advantages, challenges, and future perspectives. Cells 13:504. doi: 10.3390/cells13060504, PMID: 38534348 PMC10969765

[ref12] Caspar Y. Recule C. Pouzol P. Lafeuillade B. Mallaret M. R. Maurin M.. (2013). *Psychrobacter arenosus* bacteremia after blood transfusion, France. France. Emerg. Infect. Dis. 19, 1118–1120. doi: 10.3201/eid1907.121599, PMID: 23764120 PMC3713977

[ref13] Chen P. Jiang J. Zhang S. Wang X. Guo X. Li F. (2023). Enzymatic response and antibiotic resistance gene regulation by microbial fuel cells to resist sulfamethoxazole. Chemosphere 325:138410. doi: 10.1016/j.chemosphere.2023.138410, PMID: 36925019

[ref14] Cook M. A. Wright G. D. (2022). The past, present, and future of antibiotics. Sci. Transl. Med. 14:eabo7793. doi: 10.1126/scitranslmed.abo7793 35947678

[ref15] Corretto E. Antonielli L. Sessitsch A. Höfer C. Puschenreiter M. Widhalm S.. (2020). Comparative genomics of *Microbacterium* species to reveal diversity, potential for secondary metabolites and heavy metal resistance. Front. Microbiol. 11:1869. doi: 10.3389/fmicb.2020.01869, PMID: 32903828 PMC7438953

[ref16] Cuadrat R. R. C. Sorokina M. Andrade B. G. Goris T. Dávila A. M. R. (2020). Global Ocean resistome revealed: exploring antibiotic resistance gene abundance and distribution in TARA oceans samples. Gigascience 9, 1–12. doi: 10.1093/gigascience/giaa046 PMC7213576 32391909

[ref17] Darby E. M. Trampari E. Siasat P. Gaya M. S. Alav I. Webber M. A.. (2023). Molecular mechanisms of antibiotic resistance revisited. Nat. Rev. Microbiol. 21, 280–295. doi: 10.1038/s41579-022-00820-y, PMID: 36411397

[ref18] Đukanović S. Ganić T. Lončarević B. Cvetković S. Nikolić B. Tenji D.. (2022). Elucidating the antibiofilm activity of Frangula emodin against *Staphylococcus aureus* biofilms. J. Appl. Microbiol. 132, 1840–1855. doi: 10.1111/jam.15360 34779074

[ref19] Esposito S. Russo E. De Simone G. Gioia R. Noviello S. Vitolo M.. (2015). Transient bacteraemia due to *Chryseobacterium indologenes* in an immunocompetent patient: a case report and literature review. J. Chemother. 27, 324–329. doi: 10.1179/1973947814Y.0000000206, PMID: 25096711

[ref20] Felis E. Sochacki A. Bajkacz S. Łuczkiewicz A. Jóźwiakowski K. García J.. (2024). Removal of selected sulfonamides and sulfonamide resistance genes from wastewater in full-scale constructed wetlands. Sci. Total Environ. 912:169195. doi: 10.1016/j.scitotenv.2023.169195, PMID: 38081427

[ref21] Fisher J. F. Mobashery S. (2016). Β-Lactam resistance mechanisms: gram-positive bacteria and *Mycobacterium tuberculosis*. Cold Spring Harb. Perspect. Med. 6:a025221. doi: 10.1101/cshperspect.a025221 27091943 PMC4852796

[ref22] Galler H. Feierl G. Petternel C. Reinthaler F. F. Haas D. Habib J.. (2018). Multiresistant bacteria isolated from activated sludge in Austria. Int. J. Environ. Res. Public Health 15:479. doi: 10.3390/ijerph15030479 29522474 PMC5877024

[ref23] Garneau-Tsodikova S. Labby K. J. (2016). Mechanisms of resistance to aminoglycoside antibiotics: overview and perspectives. Med. Chem. Commun. 7, 11–27. doi: 10.1039/C5MD00344J, PMID: 26877861 PMC4752126

[ref24] Ha D.-G. Kuchma S. L. O’Toole G. A. (2014). Plate-based assay for swimming motility in *Pseudomonas aeruginosa*. Methods Mol. Biol. 1149, 59–65. doi: 10.1007/978-1-4939-0473-0_7, PMID: 24818897 PMC9007281

[ref25] Hamdaoui N. Benkirane C. Bouaamali H. Azghar A. Mouncif M. Maleb A.. (2024). Investigating lactic acid bacteria genus *Lactococcus lactis* properties: antioxidant activity, antibiotic resistance, and antibacterial activity against multidrug-resistant bacteria *Staphylococcus aureus*. Heliyon 10:e31957. doi: 10.1016/j.heliyon.2024.e31957, PMID: 38867975 PMC11168319

[ref26] Hawas J. Serry F. Belal E. Gad W. Askoura M. (2024). Partial purification and characterization of a kerationolytic enzyme from *psychrobacter pulmonis* and its application. J. Microbiol. Biotechnol. Food Sci. 14:e11178. doi: 10.55251/jmbfs.11178

[ref27] Hussain H. I. Aqib A. I. Seleem M. N. Shabbir M. A. Hao H. Iqbal Z.. (2021). Genetic basis of molecular mechanisms in β-lactam resistant gram-negative bacteria. Microb. Pathog. 158:105040. doi: 10.1016/j.micpath.2021.105040, PMID: 34119627 PMC8445154

[ref28] Ioannou P. Ziogou A. Giannakodimos A. Giannakodimos I. Tsantes A. G. Samonis G. (2025). *Psychrobacter* infections in humans—a narrative review of reported cases. Antibiotics 14:140. doi: 10.3390/antibiotics14020140, PMID: 40001384 PMC11851457

[ref29] Jałowiecki Ł. Hubeny J. Harnisz M. Płaza G. (2022). Seasonal and technological shifts of the WHO priority multi-resistant pathogens in municipal wastewater treatment plant and its receiving surface water: a case study. Int. J. Environ. Res. Public Health 19:336. doi: 10.3390/ijerph19010336 PMC8751097 35010596

[ref30] Judan Cruz K. G. Takumi O. Bongulto K. A. Gandalera E. E. Kagia N. Watanabe K. (2024). Natural compound-induced downregulation of antimicrobial resistance and biofilm-linked genes in wastewater *Aeromonas* species. Front. Cell. Infect. Microbiol. 14:1456700. doi: 10.3389/fcimb.2024.1456700, PMID: 39469451 PMC11513397

[ref31] Kearns D. B. (2010). A field guide to bacterial swarming motility. Nat. Rev. Microbiol. 8, 634–644. doi: 10.1038/nrmicro2405, PMID: 20694026 PMC3135019

[ref32] Kernodle D. S. (2006). “Mechanisms of resistance to β-lactam antibiotics” in Gram-positive pathogens. eds. Fischetti V. A. Novick R. P. Ferretti J. J. Portnoy D. A. Rood J. I. (Washington, DC: American Society for Microbiology), 769–781.

[ref33] Khemariya P. Singh S. Nath G. Gulati A. K. (2017). Probiotic *Lactococcus lactis*: a review. Turkish J. Agricul. Food Sci. Technol. 5, 556–562. doi: 10.24925/turjaf.v5i6.556-562.690

[ref34] Kim D.-W. Heinze T. M. Kim B.-S. Schnackenberg L. K. Woodling K. A. Sutherland J. B. (2011). Modification of norfloxacin by a *Microbacterium* sp. strain isolated from a wastewater treatment plant. Appl. Environ. Microbiol. 77, 6100–6108. doi: 10.1128/AEM.00545-11 21724893 PMC3165385

[ref35] Kirby J. T. Sader H. S. Walsh T. R. Jones R. N. (2004). Antimicrobial susceptibility and epidemiology of a worldwide collection of *Chryseobacterium* spp.: report from the SENTRY antimicrobial surveillance program (1997-2001). J. Clin. Microbiol. 42, 445–448. doi: 10.1128/jcm.42.1.445-448.2004 14715802 PMC321713

[ref36] Kumar S. Stecher G. Li M. Knyaz C. Tamura K. (2018). Molecular evolutionary genetics analysis across computing platforms. Mol. Biol. Evol. 35, 1547–1549. doi: 10.1093/molbev/msy096 29722887 PMC5967553

[ref37] Lasa A. Romalde J. L. (2017). Genome sequence of three *Psychrobacter* sp. strains with potential applications in bioremediation. Genomics Data 12, 7–10. doi: 10.1016/j.gdata.2017.01.005, PMID: 28229046 PMC5312645

[ref38] Lawe-Davies O. Bennett S. (2017). WHO–list of bacteria for which new antibiotics are urgently needed: WHO Department of Communications.

[ref39] Learman D. R. Ahmad Z. Brookshier A. Henson M. W. Hewitt V. Lis A.. (2019). Comparative genomics of 16 *Microbacterium* spp. that tolerate multiple heavy metals and antibiotics. PeerJ 6:e6258. doi: 10.7717/peerj.6258, PMID: 30671291 PMC6336093

[ref40] Lee J.-H. Kim Y.-G. Yong Ryu S. Lee J. (2016). Calcium-chelating alizarin and other anthraquinones inhibit biofilm formation and the hemolytic activity of *Staphylococcus aureus*. Sci. Rep. 6:19267. doi: 10.1038/srep19267, PMID: 26763935 PMC4725881

[ref41] Li S. Ondon B. S. Ho S.-H. Jiang J. Li F. (2022). Antibiotic resistant bacteria and genes in wastewater treatment plants: from occurrence to treatment strategies. Sci. Total Environ. 838:156544. doi: 10.1016/j.scitotenv.2022.156544, PMID: 35679932

[ref42] Li L. Song X. Yin Z. Jia R. Li Z. Zhou X.. (2016). The antibacterial activity and action mechanism of emodin from *Polygonum cuspidatum* against *Haemophilus parasuis in vitro*. Microbiol. Res. 186-187, 139–145. doi: 10.1016/j.micres.2016.03.008, PMID: 27242151

[ref43] Liang X. Zhang J. Kim Y. Ho J. Liu K. Keenum I.. (2023). ARGem: a new metagenomics pipeline for antibiotic resistance genes: metadata, analysis, and visualization. Front. Genet. 14:1219297. doi: 10.3389/fgene.2023.1219297, PMID: 37811141 PMC10558085

[ref44] Luthra S. Rominski A. Peter S. (2018). The role of antibiotic-target-modifying and antibiotic-modifying enzymes in *Mycobacterium abscessus* drug resistance. Front. Microbiol. 9:2179. doi: 10.3389/fmicb.2018.02179 30258428 PMC6143652

[ref45] Magiorakos A. P. Srinivasan A. Carey R. B. Carmeli Y. Falagas M. E. Giske C. G.. (2012). Multidrug-resistant, extensively drug-resistant and pandrug-resistant bacteria: an international expert proposal for interim standard definitions for acquired resistance. Clin. Microbiol. Infect. 18, 268–281. doi: 10.1111/j.1469-0691.2011.03570.x, PMID: 21793988

[ref46] Matu A. Lum Nde A. Oosthuizen L. Hitzeroth A. Badenhorst M. Duba L.. (2019). Draft genome sequences of seven *Chryseobacterium* type strains. Microbiol. Resour. Announc. 8:e01518. doi: 10.1128/mra.01518-18, PMID: 30637405 PMC6318376

[ref47] McEwen S. A. Collignon P. J. (2018). Antimicrobial resistance: a one health perspective. Microbiol. Spectr. 6, 521–547. doi: 10.1128/9781555819804.ch25 PMC11633550 29600770

[ref48] Mojia P. L. (2017). Prioritization of pathogens to guide discovery, research and development of new antibiotics for drug resistant bacterial infections, including tuberculosis World Health Organization

[ref49] Mondal A. H. Khare K. Saxena P. Debnath P. Mukhopadhyay K. Yadav D. (2024). A review on colistin resistance: an antibiotic of last resort. Microorganisms 12:772. doi: 10.3390/microorganisms12040772, PMID: 38674716 PMC11051878

[ref50] Naiel M. A. El-Kholy A. I. Negm S. S. Ghazanfar S. Shukry M. Zhang Z.. (2023). A mini-review on plant-derived phenolic compounds with particular emphasis on their possible applications and beneficial uses in aquaculture. Ann. Anim. Sci. 23, 971–977. doi: 10.2478/aoas-2023-0007

[ref51] Nguyen T. L. A. Bhattacharya D. (2022). Antimicrobial activity of quercetin: an approach to its mechanistic principle. Molecules 27:2494. doi: 10.3390/molecules27082494, PMID: 35458691 PMC9029217

[ref52] Oldham A. L. Duncan K. E. (2012). Similar gene estimates from circular and linear standards in quantitative PCR analyses using the prokaryotic 16S rRNA gene as a model. PLoS One 7:e51931. doi: 10.1371/journal.pone.0051931, PMID: 23284822 PMC3526484

[ref53] OneHealthTrust (2023). Resistancemap: antibiotic use. Available online at: https://resistancemap.onehealthtrust.org/AntibioticResistance.php

[ref54] Ozaktas T. Taskin B. Gozen A. G. (2012). High level multiple antibiotic resistance among fish surface associated bacterial populations in non-aquaculture freshwater environment. Water Res. 46, 6382–6390. doi: 10.1016/j.watres.2012.09.010, PMID: 23039919

[ref55] Pan X. S. Fisher L. M. (1997). Targeting of DNA gyrase in *Streptococcus pneumoniae* by sparfloxacin: selective targeting of gyrase or topoisomerase IV by quinolones. Antimicrob. Agents Chemother. 41, 471–474. doi: 10.1128/aac.41.2.471, PMID: 9021211 PMC163733

[ref56] Partridge S. R. Kwong S. M. Firth N. Jensen O. (2018). Mobile genetic elements associated with antimicrobial resistance. Clin. Microbiol. Rev. 31:e00088-17. doi: 10.1128/cmr.00088-17 30068738 PMC6148190

[ref57] Pham D. N. Li M. (2024). Comparative resistomics analysis of multidrug-resistant *Chryseobacteria*. Environ. Microbiol. Rep. 16:e13288. doi: 10.1111/1758-2229.13288, PMID: 38923192 PMC11194056

[ref58] Phuong Hoa P. T. Nonaka L. Hung Viet P. Suzuki S. (2008). Detection of the *sul1*, *sul2*, and *sul3* genes in sulfonamide-resistant bacteria from wastewater and shrimp ponds of North Vietnam. Sci. Total Environ. 405, 377–384. doi: 10.1016/j.scitotenv.2008.06.023, PMID: 18684492

[ref59] Prestinaci F. Pezzotti P. Pantosti A. (2015). Antimicrobial resistance: a global multifaceted phenomenon. Pathog. Glob. Health 109, 309–318. doi: 10.1179/2047773215Y.0000000030, PMID: 26343252 PMC4768623

[ref60] Rizzo L. Manaia C. Merlin C. Schwartz T. Dagot C. Ploy M. C.. (2013). Urban wastewater treatment plants as hotspots for antibiotic resistant bacteria and genes spread into the environment: a review. Sci. Total Environ. 447, 345–360. doi: 10.1016/j.scitotenv.2013.01.032, PMID: 23396083

[ref61] Roberts M. C. Schwarz S. (2017). “Tetracycline and chloramphenicol resistance mechanisms” in Antimicrobial drug resistance: Mechanisms of drug resistance. eds. Mayers D. L. Sobel J. D. Ouellette M. Kaye K. S. Marchaim D., vol. 1 (Cham: Springer International Publishing), 231–243.

[ref62] Rodrigues D. F. Ayala-del-Río H. Pellizari V. Gilichinsky D. Sepulveda-Torres L. Tiedje J. (2009). Biogeography of two cold-adapted genera: *Psychrobacter* and *Exiguobacterium*. ISME J. 3, 658–665. doi: 10.1038/ismej.2009.25, PMID: 19322243

[ref63] Rout A. K. Tripathy P. S. Dixit S. Behera D. U. Behera B. Das B. K.. (2024). Metagenomics analysis of sediments of river Ganga, India for bacterial diversity, functional genomics, antibiotic resistant genes and virulence factors. Curr. Res. Biotechnol. 7:100187. doi: 10.1016/j.crbiot.2024.100187, PMID: 40396621

[ref64] Santacroce L. Topi S. Charitos I. A. Lovero R. Luperto P. Palmirotta R.. (2024). Current views about the inflammatory damage triggered by bacterial superantigens and experimental attempts to neutralize superantigen-mediated toxic effects with natural and biological products. Pathophysiology 31, 18–31. doi: 10.3390/pathophysiology31010002, PMID: 38251046 PMC10801599

[ref65] Saticioglu I. B. Duman M. Altun S. (2021). Genome analysis and antimicrobial resistance characteristics of *Chryseobacterium aquaticum* isolated from farmed salmonids. Aquaculture 535:736364. doi: 10.1016/j.aquaculture.2021.736364

[ref66] Sauer K. Stoodley P. Goeres D. M. Hall-Stoodley L. Burmølle M. Stewart P. S.. (2022). The biofilm life cycle: expanding the conceptual model of biofilm formation. Nat. Rev. Microbiol. 20, 608–620. doi: 10.1038/s41579-022-00767-0, PMID: 35922483 PMC9841534

[ref67] Singh A. Pratap S. G. Raj A. (2024). Occurrence and dissemination of antibiotics and antibiotic resistance in aquatic environment and its ecological implications: a review. Environ. Sci. Pollut. Res. 31, 47505–47529. doi: 10.1007/s11356-024-34355-x, PMID: 39028459

[ref68] Sodhi K. K. Kumar M. Dhaulaniya A. S. Balan B. Singh D. K. (2021). Enhanced ciprofloxacin removal by plant growth-promoting *Microbacterium* sp. WHC1 in presence of *Eichhornia crassipes* root exudates. Environ. Sustain. 4, 143–153. doi: 10.1007/s42398-020-00153-7

[ref69] Solano-Gálvez S. G. Valencia-Segrove M. F. Prado M. J. O. Boucieguez A. B. L. Álvarez-Hernández D. A. Vázquez-López R. (2020). “Mechanisms of resistance to quinolones” in Antimicrobial resistance-a one health perspective. eds. Mares M. Lim S. H. E. Lai K.-S. Cristina R.-T. (London: IntechOpen), 25–48.

[ref70] Soni K. Jyoti K. Chandra H. Chandra R. (2022). Bacterial antibiotic resistance in municipal wastewater treatment plant; mechanism and its impacts on human health and economy. Bioresour. Technol. Rep. 19:101080. doi: 10.1016/j.biteb.2022.101080

[ref71] Stewart P. S. (2002). Mechanisms of antibiotic resistance in bacterial biofilms. Int. J. Med. Microbiol. 292, 107–113. doi: 10.1078/1438-4221-00196, PMID: 12195733

[ref72] Surleac M. Czobor Barbu I. Paraschiv S. Popa L. I. Gheorghe I. Marutescu L.. (2020). Whole genome sequencing snapshot of multi-drug resistant *Klebsiella pneumoniae* strains from hospitals and receiving wastewater treatment plants in southern Romania. PLoS One 15:e0228079. doi: 10.1371/journal.pone.0228079, PMID: 31999747 PMC6992004

[ref73] Tyagi P. Singh M. Kumari H. Kumari A. Mukhopadhyay K. (2015). Bactericidal activity of curcumin I is associated with damaging of bacterial membrane. PLoS One 10:e0121313. doi: 10.1371/journal.pone.0121313, PMID: 25811596 PMC4374920

[ref74] Uluseker C. Kaster K. M. Thorsen K. Basiry D. Shobana S. Jain M.. (2021). A review on occurrence and spread of antibiotic resistance in wastewaters and in wastewater treatment plants: mechanisms and perspectives. Front. Microbiol. 12:717809. doi: 10.3389/fmicb.2021.717809, PMID: 34707579 PMC8542863

[ref75] Uyaguari-Díaz M. I. Croxen M. A. Luo Z. Cronin K. I. Chan M. Baticados W. N.. (2018). Human activity determines the presence of integron-associated and antibiotic resistance genes in southwestern British Columbia. Front. Microbiol. 9:852. doi: 10.3389/fmicb.2018.00852, PMID: 29765365 PMC5938356

[ref76] Wan X. Takala T. M. Qiao M. Saris P. E. (2021). Complete genome sequence of nisin-producing *Lactococcus lactis* subsp. *lactis* N8. Microbiol. Resour. Announc. 10:e01147-20. doi: 10.1128/mra.01147-20 33414301 PMC8407701

[ref77] WHO (2017). Global antimicrobial resistance surveillance system (GLASS) report: early implementation 2016-2017. Available at: https://pesquisa.bvsalud.org/portal/resource/pt/who-259744

[ref78] Wozniak T. M. Barnsbee L. Lee X. J. Pacella R. E. (2019). Using the best available data to estimate the cost of antimicrobial resistance: a systematic review. Antimicrob. Resist. Infect. Control 8:26. doi: 10.1186/s13756-019-0472-z, PMID: 30733860 PMC6359818

[ref79] Wu Y. Li S. Yu K. Hu J. Chen Q. Sun W. (2023). Wastewater treatment plant effluents exert different impacts on antibiotic resistome in water and sediment of the receiving river: metagenomic analysis and risk assessment. J. Hazard. Mater. 460:132528. doi: 10.1016/j.jhazmat.2023.132528, PMID: 37713776

[ref80] Xiu P. Liu R. Zhang D. Sun C. (2017). Pumilacidin-like lipopeptides derived from marine bacterium *Bacillus* sp. strain 176 suppress the motility of *Vibrio alginolyticus*. Appl. Environ. Microbiol. 83:e00450-17. doi: 10.1128/AEM.00450-17 28389538 PMC5452807

[ref81] Yu X. Li Z. Yang X. Wang X. Nan D. Lu W.. (2023). *Microbacterium* spp. peritonitis in patients undergoing peritoneal dialysis: a single-center experience and literature review. Front. Med. 10:1297296. doi: 10.3389/fmed.2023.1297296, PMID: 38076234 PMC10701896

[ref82] Yun M.-K. Wu Y. Li Z. Zhao Y. Waddell M. B. Ferreira A. M.. (2012). Catalysis and sulfa drug resistance in dihydropteroate synthase. Science 335, 1110–1114. doi: 10.1126/science.1214641, PMID: 22383850 PMC3531234

[ref83] Zárate S. G. Bastida A. Santana A. G. Revuelta J. (2019). Synthesis of ring II/III fragment of kanamycin: a new minimum structural motif for aminoglycoside recognition. Antibiotics 8:109. doi: 10.3390/antibiotics8030109, PMID: 31382490 PMC6783941

[ref84] Zheng D. Huang C. Huang H. Zhao Y. Khan M. R. U. Zhao H.. (2020). Antibacterial mechanism of curcumin: a review Chem. Biodivers 17:e2000171. doi: 10.1002/cbdv.202000171 32533635

