# Supplementary material for: From wastewater to resistance: characterization of multidrug-resistant bacteria and assessment of natural antimicrobial compounds
Source: Front Microbiol. 2025 Jul 10;16:1612534. doi: 10.3389/fmicb.2025.1612534 (PMC12286950; doi:10.3389/fmicb.2025.1612534)
Supplement: Supplementary file 1 [file Supplementary_file_1.docx]

**Natural Compounds as Antimicrobial Agents Against Multi-Drug Resistant Bacteria from Urban Wastewater Effluent**

Mingyue Li^1,2,3^, Angela Zhan^1,2,4^, Tahira Rahman^1,2^, Tao Jiang^5^, Liyuan Hou^1,2*^

1 Utah Water Research Laboratory, 1600 Canyon Road, Logan, UT 84321, United States

2 Department of Civil and Environmental Engineering, Utah State University, Logan, UT 84322, United States

3 School of Resources and Environmental Engineering, Shandong University of Technology, Zibo 255049, China

4 Logan High School, Logan, UT 84321, USA

5 Department of Environmental and Sustainable Engineering, University at Albany, State University of New York, Albany, New York 12222, United States

***Correspondence to:**

Dr. Liyuan Hou; Email: [liyuan.hou@usu.edu](mailto:liyuan.hou@usu.edu); Tel: 1-435-797-1533

Mingyue Li and Angela Zhan contributed equally to this work.

**Table S1.** Characteristics of nine isolates.

| Isolate name | Nearest Taxonomic Neighbor | Identity | Phylum | Genus |
| --- | --- | --- | --- | --- |
| U1 | *Microbacterium oxydans strain M90* | 99.60% | *Actinobacteria* | *Microbacterium* |
|  | *Microbacterium maritypicum strain Snea159* | 99.46% | *Actinobacteria* | *Microbacterium* |
| U2 | *Microbacterium maritypicum strain Snea159* | 99.13% | *Actinobacteria* | *Microbacterium* |
|  | *Microbacterium oxydans strain ML-6* | 99.02% | *Actinobacteria* | *Microbacterium* |
| U3 | *Chryseobacterium sp. HP3E* | 99.34% | *Bacteroidetes* | *Chryseobacterium* |
|  | *Chryseobacterium sp. PDD-58b-7* | 99.12% | *Bacteroidetes* | *Chryseobacterium* |
| U4 | *Lactococcus lactis subsp. lactis IO-1* | 99.74% | *Firmicutes* | *Lactococcus lactis* |
|  | *Lactococcus lactis subsp. lactis strain A12* | 99.74% | *Firmicutes* | *Lactococcus lactis* |
| U5 | *Chryseobacterium sp. HP3E* | 99.34% | *Bacteroidetes* | *Chryseobacterium* |
|  | *Epilithonimonas sp. PDD-58b-7* | 99.12% | *Firmicutes* | *Epilithonimonas* |
| U6 | *Lactococcus lactis strain HBUAS58280* | 100.00% | *Firmicutes* | *Lactococcus lactis* |
|  | *Lactococcus lactis strain 4355* | 100.00% | *Firmicutes* | *Lactococcus lactis* |
| U7 | *Chryseobacterium sp. 5127* | 99.80% | *Bacteroidetes* | *Chryseobacterium* |
|  | *Chryseobacterium aquaticum strain KR2-2* | 99.59% | *Bacteroidetes* | *Chryseobacterium* |
| U8 | *Lactococcus lactis strain HBUAS58280* | 99.86% | *Firmicutes* | *Lactococcus lactis* |
|  | *Lactococcus lactis strain Mise173* | 99.86% | *Firmicutes* | *Lactococcus lactis* |
| U9 | *Psychrobacter pulmonis strain MB36* | 100.00% | *Proteobacteria* | *Psychrobacter* |
|  | *Psychrobacter sp. BSw20884b* | 100.00% | *Proteobacteria* | *Psychrobacter* |

**Table S2**. WHO priority pathogens list for research and development of new antibiotics

| Categories | Bacteria | Antibiotic Resistance |
| --- | --- | --- |
| Critical | *Acinetobacter baumannii* | carbapenem-resistant |
|  | *Pseudomonas aeruginosa* | carbapenem-resistant |
|  | Enterobacteriaceae | carbapenem-resistant,  ESBL-producing |
| High | *Enterococcus faecium* | vancomycin-resistant |
|  | *Staphylococcus aureus* | methicillin-resistant,  vancomycin-intermediate and resistant |
|  | *Helicobacter pylori* | clarithromycin-resistant |
|  | *Campylobacter spp.* | fluoroquinolone-resistant |
|  | *Salmonellae spp.* | fluoroquinolone-resistant |
|  | *Neisseria gonorrhoeae* | cephalosporin-resistant,  fluoroquinolone-resistant |
| Medium | *Streptococcus pneumoniae* | penicillin-non-susceptible |
|  | *Haemophilus influenzae* | ampicillin-resistant |
|  | *Shigella spp.* | fluoroquinolone-resistant |


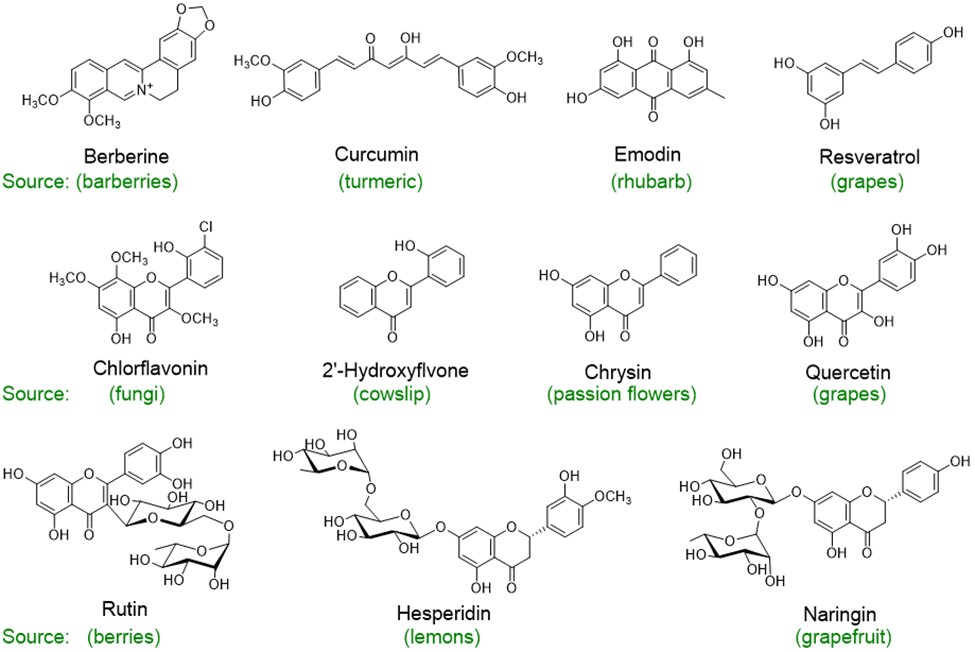


**Figure S1.** Structures and representative sources of the screened natural compounds.


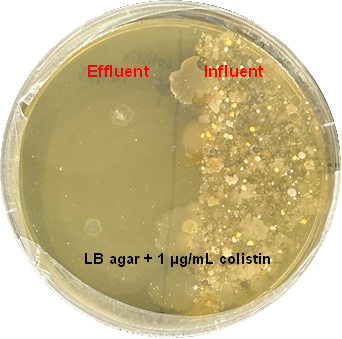


**Figure S2.** Testing of the tolerance of influent and effluent microbes to colistin.


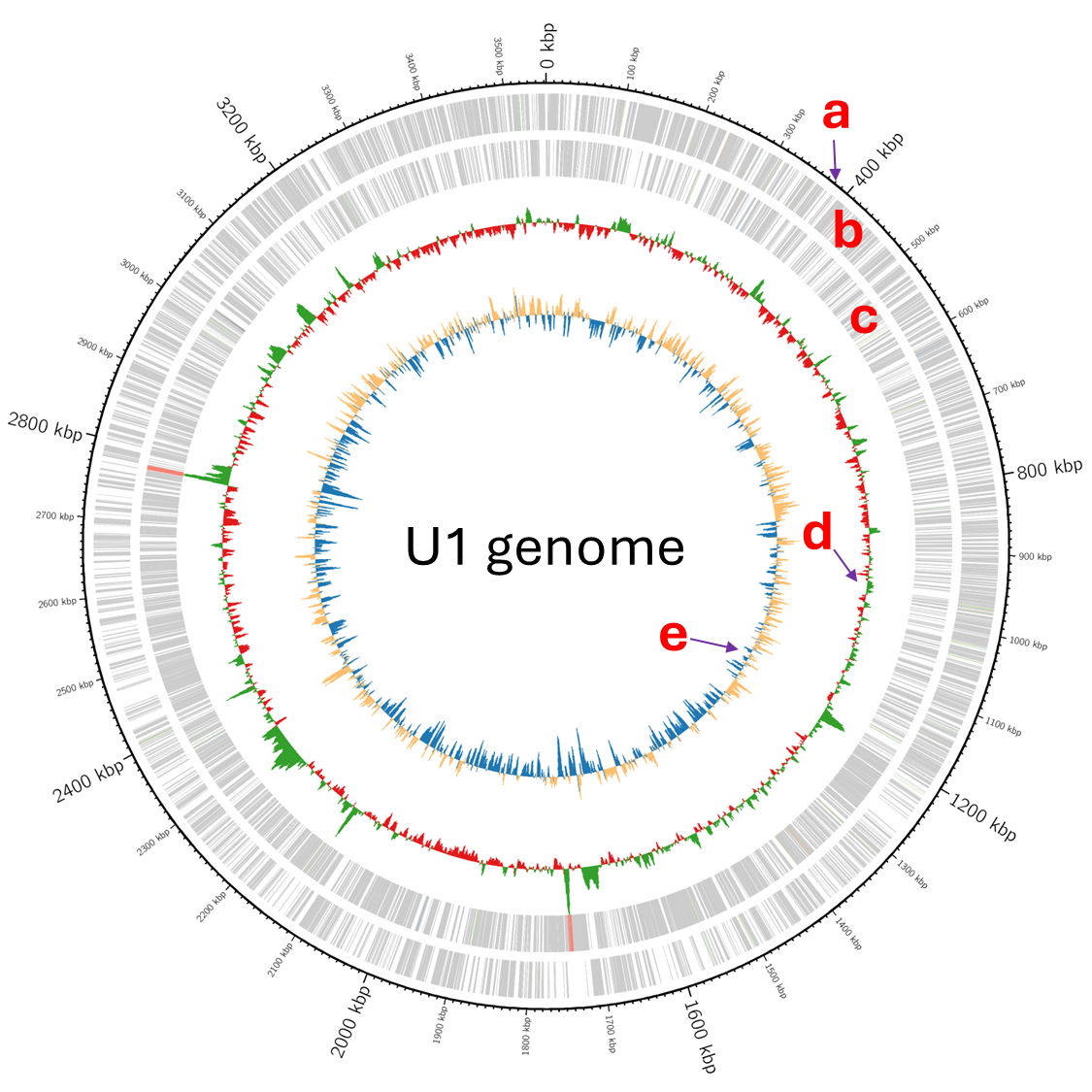

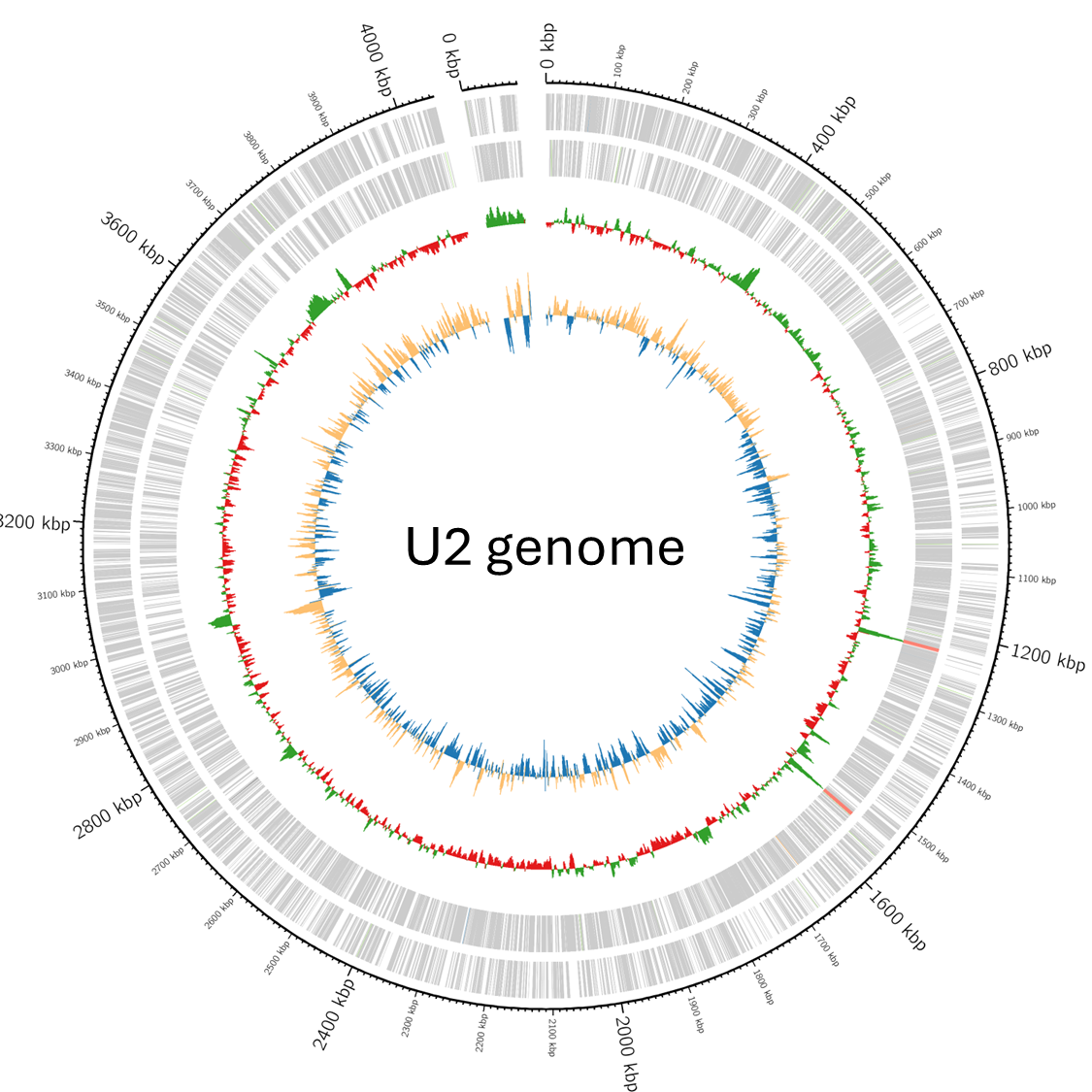

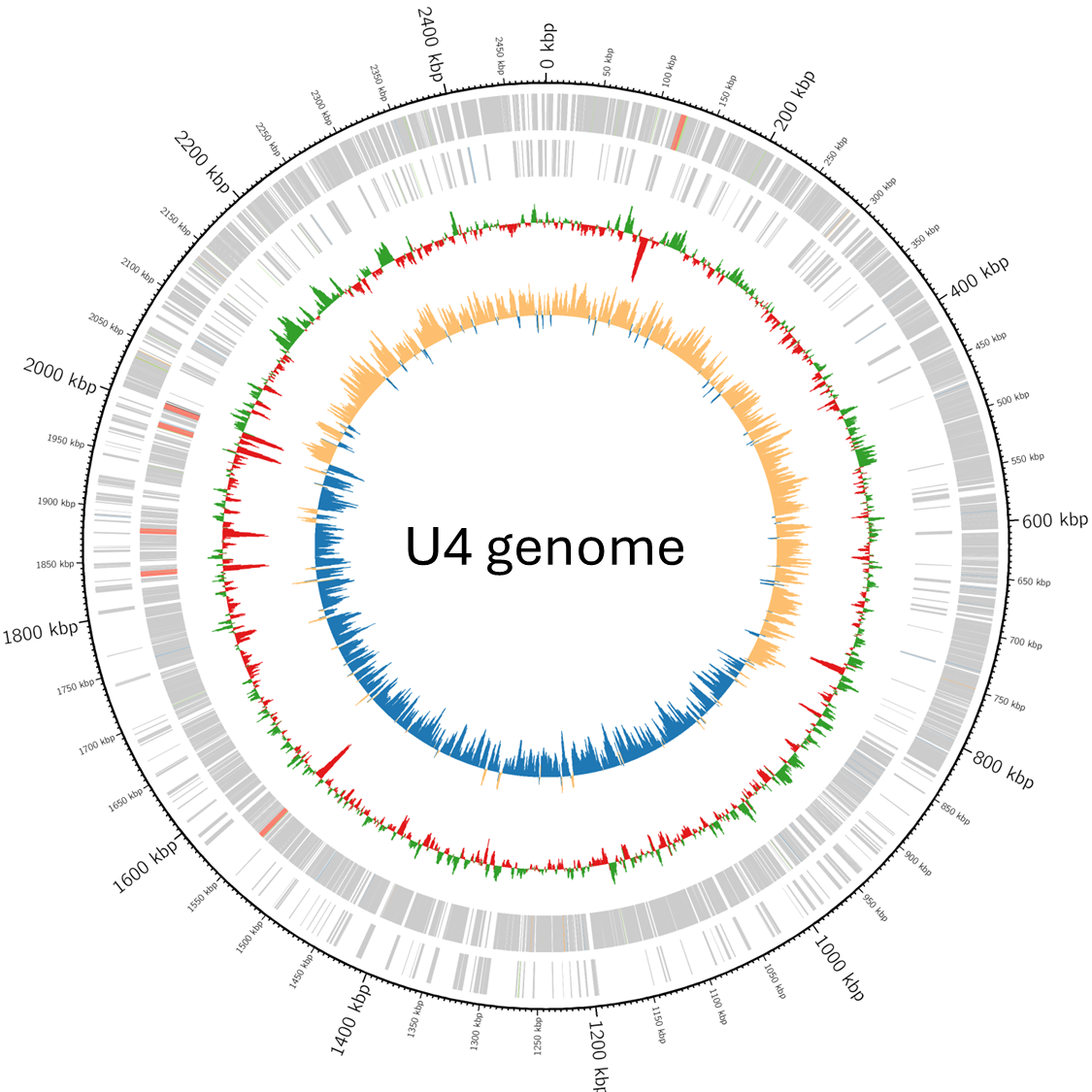

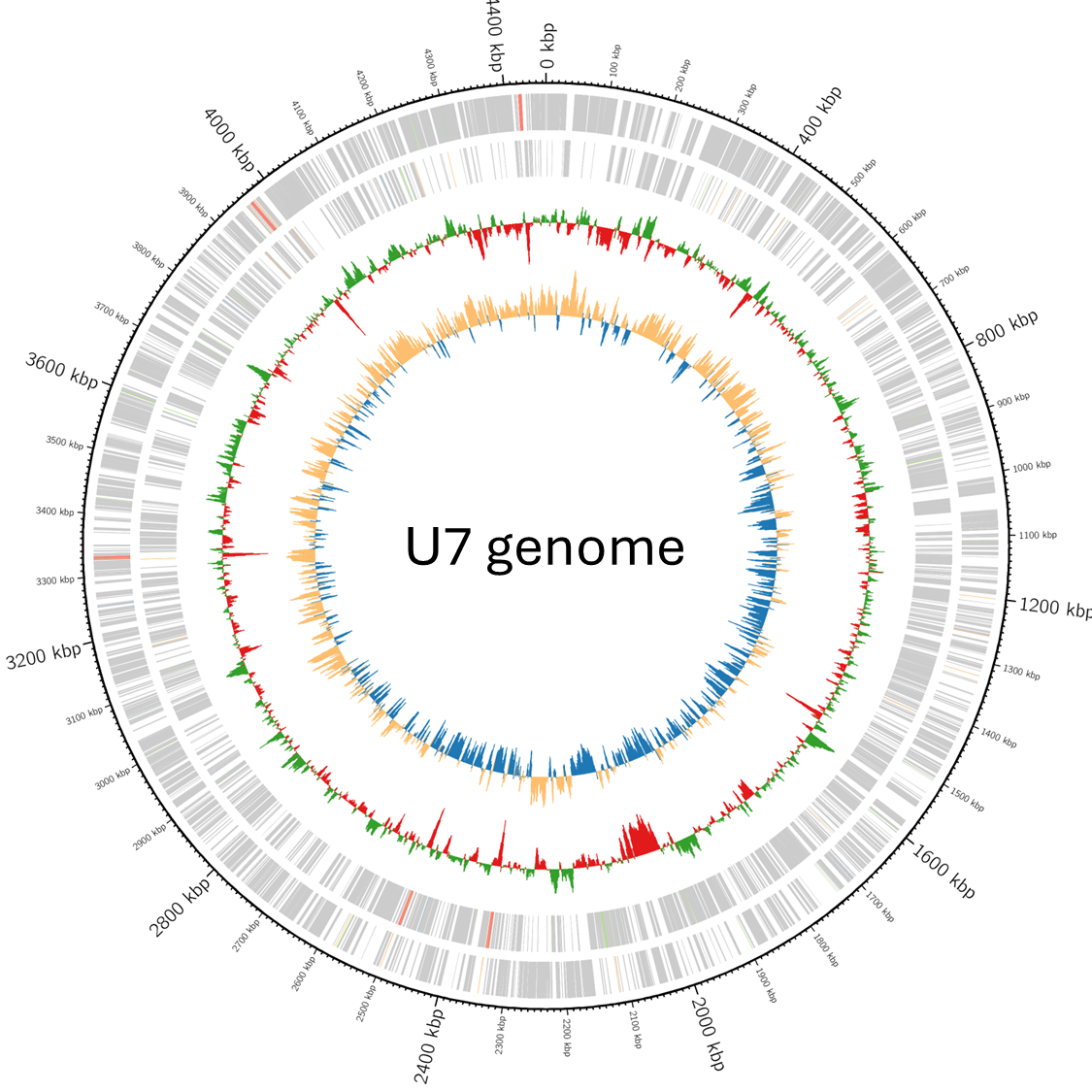


A

B

C

D

**Figure S3**. Genome maps of U1 (A), U2 (B), U4 (C) and U7 (D). a: Genome size; b: Coding sequences in the forward strand; c: coding sequences in the reverse strand; d: GC skew metric; e: GC ratio metric.


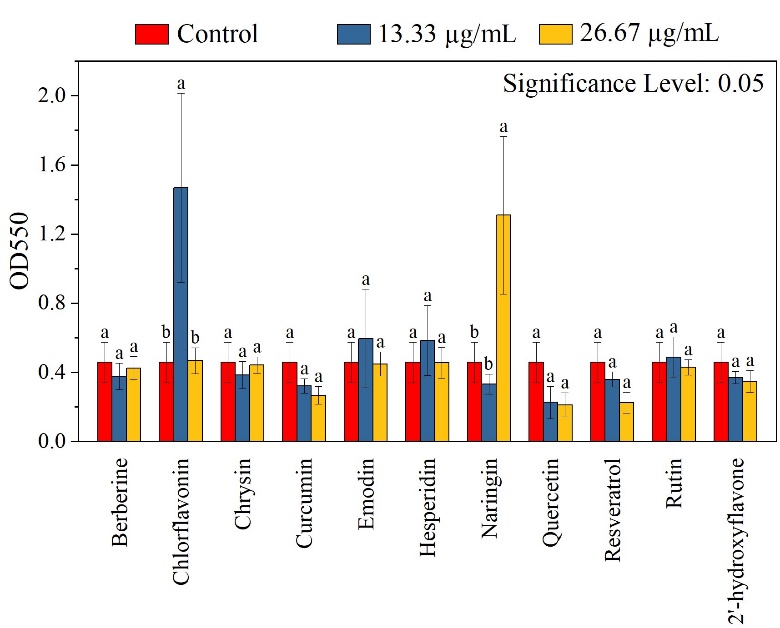


**Figure S4.** The effect of 11 natural compounds on biofilm formation of *Pseudomonas aeruginosa* PAO1. The variation between different treatments was conducted by a paired comparison plot. Different letters indicate significant at 0.05 level.
